# Impacts of local population history and ecology on the evolution of a globally dispersed pathogen

**DOI:** 10.1186/s12864-020-06778-6

**Published:** 2020-05-20

**Authors:** Andreina I. Castillo, Carlos Chacón-Díaz, Neysa Rodríguez-Murillo, Helvecio D. Coletta-Filho, Rodrigo P. P. Almeida

**Affiliations:** 1 Department of Environmental Science, Policy and Management, University of California, Berkeley, CA USA; 2 Centro de Investigación en Enfermedades Tropicales, Facultad de Microbiología, Universidad de Costa Rica, San José, Costa Rica; 3 Centro de Citricultura Sylvio Moreira, IAC, C.P. 4, Cordeirópolis, SP 13490-970 Brazil

**Keywords:** *Xylella fastidiosa*, WGS, Inter-subspecific recombination, Genetic diversity, Pan genome

## Abstract

**Background:**

Pathogens with a global distribution face diverse biotic and abiotic conditions across populations. Moreover, the ecological and evolutionary history of each population is unique. *Xylella fastidiosa* is a xylem-dwelling bacterium infecting multiple plant hosts, often with detrimental effects. As a group, *X. fastidiosa* is divided into distinct subspecies with allopatric historical distributions and patterns of multiple introductions from numerous source populations. The capacity of *X. fastidiosa* to successfully colonize and cause disease in naïve plant hosts varies among subspecies, and potentially, among populations. Within Central America (i.e. Costa Rica) two *X. fastidiosa* subspecies coexist: the native subsp. *fastidiosa* and the introduced subsp. *pauca*. Using whole genome sequences, the patterns of gene gain/loss, genomic introgression, and genetic diversity were characterized within Costa Rica and contrasted to other *X. fastidiosa* populations.

**Results:**

Within Costa Rica, accessory and core genome analyses showed a highly malleable genome with numerous intra- and inter-subspecific gain/loss events. Likewise, variable levels of inter-subspecific introgression were found within and between both coexisting subspecies; nonetheless, the direction of donor/recipient subspecies to the recombinant segments varied. Some strains appeared to recombine more frequently than others; however, no group of genes or gene functions were overrepresented within recombinant segments. Finally, the patterns of genetic diversity of subsp. *fastidiosa* in Costa Rica were consistent with those of other native populations (i.e. subsp. *pauca* in Brazil).

**Conclusions:**

Overall, this study shows the importance of characterizing local evolutionary and ecological history in the context of world-wide pathogen distribution.

## Background

In plant pathology, three major components are considered key in the development of plant disease: *(i)* the environment must be suitable for disease symptom expression; *(ii)* plant hosts need to be susceptible to infection; and *(iii)* pathogens must be virulent [1]. In most cases however, plant interactions with microorganisms are not pathogenic. What then, are the combined ecological and evolutionary events leading to the development of disease in plants? And how do the evolutionary and ecological events acting within a population, isolated or not, influence the evolution of an entire species? To address these questions, a better understanding of the evolutionary and ecological history of individual populations is crucial [2], especially in the context of globally spread pathogens.

The diversity of bacterial pathogens makes them ideal models to evaluate these topics. Detailed studies in human colonizing bacteria have led to comprehensive descriptions of their evolutionary histories, epidemiologies, and the continuous risk assessment and management of many major pathogens [3–5]. However, despite the existence of numerous ecologically and economically important bacterial plant pathogens [6], similar studies are often not performed with such depth or scope. Recent studies have described the evolutionary history and ecology of diverse *Xylella fastidiosa* populations worldwide [7–12]. Each population has a unique evolutionary relationship as well as being subjected to distinct ecological forces. In this regard, *X. fastidiosa* can be adequately used to better understand the role of local evolutionary dynamics on the global spread of plant pathogens.

*X. fastidiosa* is a xylem-dwelling bacterium transmissible to multiple plant hosts by numerous species of sap-feeding insects such as sharpshooters and spittlebugs [13–15]. *X. fastidiosa* causes diverse symptoms with detrimental effects in both yield and quality of agricultural crops [16]. As a species, *X. fastidiosa* has been reported in at least 563 plant species from 82 botanical families [17]. This broad host range led to the original assumption that *X. fastidiosa* is a generalist [18]; nonetheless, later analyses showed that *X. fastidiosa*’s host range varies at the inter- [19, 20] and intra-subspecific level [21]. *X. fastidiosa* has been classified into five separate subspecies, three of which are monophyletic and ancestrally allopatric: subsp*. multiplex* (native to temperate and subtropical North America) [22, 23], subsp*. pauca* (native to South America) [23], and subsp*. fastidiosa* (native to Central America) [19]. Another recognized subspecies, subsp*. sandyi* is found in Southern regions of North America [24, 25] and has been detected in Europe [26]. The fifth named subspecies, subsp*. morus*, is not a vertically descended group and is instead believed to be the product of inter-subspecific recombination between subsp*. multiplex* and subsp*. fastidiosa* [9, 27].

*X. fastidiosa* has a complex ecological and evolutionary history. The introduction of foreign plant species to areas where *X. fastidiosa* is native, as well as the human-facilitated movement of infected plants across geographic regions, has resulted in *X. fastidiosa* outbreaks. Strong evidence shows that subsp*. fastidiosa* was introduced to the USA approximately 150 years ago [8, 11]. Likewise, subsp*. multiplex* [28] has been introduced to South America and subsp*. pauca* is proposed to have been introduced into Central America ~ 50 years ago [9]. Moreover, multiple *X. fastidiosa* subspecies have been introduced to diverse European regions from the Americas in the last few decades [7, 10, 29, 30].

The evolutionary forces and the ecological background of each of these *X. fastidiosa* populations are unique and could have different contributions to *X. fastidiosa* evolution. For instance, genetic exchange in the form of homologous recombination has been known to happen between co-occurring *X. fastidiosa* subspecies [22, 28]. A novel introduction originating from these locations might carry a different genetic background than an introduction originating from a location where a single *X. fastidiosa* subspecies exists. Similarly, introductions to locations of higher plant diversity will likely evolve differently than introductions to monocultures [31]. Therefore, to better characterize *X. fastidiosa* evolution as a group we must first explore the genomic changes occurring in each population.

Among all these geographic and chronological points, Central America -specifically Costa Rica- stands out for its evolutionary and ecological relevance to *X. fastidiosa*. Central America represents the native center of subsp. *fastidiosa*, acts as the source population for outbreaks in North America and is the putative introduction point of subsp. *pauca* from South America. Because of these attributes, a better characterization of the evolutionary forces acting on the two coexisting *X. fastidiosa* subspecies present in Costa Rica is of value in increasing our knowledge on *X. fastidiosa* overall. In specific, a close examination of diverse subsp*. fastidiosa* and subsp*. pauca* populations would allow us to compare the genetic diversity and genomic content across multiple native and introduced populations. Moreover, previous studies have shown that genetic exchange between sympatric *X. fastidiosa* subspecies readily occurs [27, 28]. Thus, this location would also permit us to assess the patterns of inter-subspecific genomic exchange between native and invasive pathogen populations. In addition, it would permit us to assess potential differences in gain/loss patterns of each subspecies within a single geographic region.

The following study aims to describe the adaptive and non-adaptive forces relevant to the evolution of subsp*. fastidiosa* and subsp*. pauca* within Costa Rica. We described this location regarding patterns of gene gain/loss, recombination, genetic diversity, and linkage disequilibrium within both subspecies. In addition, we further evaluate the hypothesis that subsp. *fastidiosa* is native to Central America and was introduced to the US from this region using whole genome data. In order to address both points we contextualize our findings within Costa Rica by comparing them to other *X. fastidiosa* populations. Overall, three main comparisons are explored: 1) between populations of the same subspecies (e.g., California, Southeastern US, Spain, Taiwan, and Costa Rica for subsp. *fastidiosa*; and Italy, Brazil, and Costa Rica for subsp. *pauca*); 2) between native populations (e.g. Costa Rica subsp. *fastidiosa* and Brazil subsp. *pauca*); and 3) between subspecies within the same geographic location (e.g. Costa Rica subsp. *fastidiosa* and subsp. *pauca*). Our main goal is to better understand the evolutionary history of *X. fastidiosa*, and the role that Costa Rica has in it.

## Methods

### Bacterial detection and isolation

Isolation attempts were done from asymptomatic plant material or plants showing mild symptoms, that were previously confirmed for *X. fastidiosa* by either indirect immunofluorescence [32], conventional PCR [33] or DAS-ELISA (following manufacturer recommendations; Agdia, Inc). Plant tissue for isolation was rinsed in tap water. Leaf petioles were excised and disinfected in 70% ethanol for 5 min, 1% sodium hypochlorite for 5 min and three rinses, 5 min each, in sterile water [21]. The tissue was ground in phosphate saline buffer (PBS). Serial dilutions 10^− 1^ and 10^− 2^ were prepared from the plant extract. 20 mL of undiluted and prepared dilutions were plated onto buffered charcoal yeast extract (BCYE) medium. Agar plates were incubated at 28 °C for 3 to 4 weeks. Plates were periodically evaluated for the presence of *X. fastidiosa*-like colonies. The recovered colonies were confirmed to be *X. fastidiosa* using immunofluorescence or conventional PCR. A single colony was selected and re-plated to assure purity of the strains and stored at − 80 °C in 20% glycerol.

### Whole-genome sequencing and assembly of *X. fastidiosa* isolates

The following study encompasses 261 *X. fastidiosa* isolates obtained from infected plants found in diverse geographic regions. The number of isolates available varied among locations: US-California (*n* = 141), Southeastern US (*n* = 9), Costa Rica (*n* = 16), Brazil (*n* = 15), Italy (*n* = 78), Spain (*n* = 3), and Taiwan (*n* = 2). These totals include both published assemblies and assemblies that were developed for this study. Except for Costa Rica (*n* = 13) and Brazil (*n* = 3), all data included in this study have been previously made publicly available. The use of genetic resources from Costa Rica was approved by the Institutional Biodiversity Committee of the University of Costa Rica (VI-1206-2017) according to the Biodiversity Law #7788 and the Convention on Biological Diversity. Detailed metadata on each assembly has been compiled on Supplementary Table 1 and the assembly statistics for new whole genome sequences is provided in Table 1.

**Table 1:** Assembly statistics of novel sequences included on this study (Illumina and PacBio). Metadata for all isolates used in the study can be found on Supplementary Table 1

Subspecies	Geographic origin	Isolate	Host plant	N50 (kb)	Read length (bp)	Genome length (bp)	Coverage (x)
*X. fastidiosa* subsp. *fastidiosa*	Costa Rica	XF68	*Psidium* spp.	80.121	178	2,714,514	117
		XF70	*Coffea* spp.	94.869	246	2,594,259	667
		XF71	*Coffea* spp.	95.502	184	2,637,686	59
		XF72	*Coffea* spp.	92.044	194	2,582,644	84
		XF73	*Coffea* spp.	100.56	181	2,606,440	140
		XF74	*Coffea* spp.	96.048	190	2,522,713	133
		XF75	*Coffea* spp.	86.72	189	2,624,678	146
		XF1090	*Coffea* spp.	88.998	147	2,673,407	142
		XF1093	*Coffea* spp.	87.187	149	2,643,654	119
		XF1094	*Vinca* spp.	104.842	148	2,634,626	111
		XF1105	*Coffea* spp.	97.706	148	2,603,724	168
		XF1110	*Vinca* spp.	80.632	147	2,605,605	136
*X. fastidiosa* subsp. *pauca*	Brazil	RAAR15 co33	*Coffea* spp.	145.445	90	2,667,270	714
		RAAR16 co13	*Coffea* spp.	98.264	90	2,740,681	663
		RAAR17 ciUb7	*Citrus sinensis*	114.674	90	2,681,548	659

Thirteen *X. fastidiosa* subsp. *fastidiosa* isolates were obtained from infected Costa Rican plants (10 coffee plants, 2 periwinkle plants, and 1 guava plant). Eight were sequenced using Illumina HiSeq2000 and five using both Illumina HiSeq2000 and PacBio. In addition, three *X. fastidiosa* isolates were obtained from infected Brazilian plants and sequenced using Illumina HiSeq2000. Samples were sequenced at the University of California, Berkeley Vincent J. Coates Genomics Sequencing Laboratory (California Institute for Quantitative Biosciences; QB3), and the Center for Genomic Sciences, Allegheny Singer Research Institute, Pittsburgh, PA. All raw reads and information regarding each strain have been submitted to the following bioprojects: PRJNA576471 (Costa Rican isolates) and PRJNA576479 (Brazilian isolates). A single Costa Rica isolate (XF69) was removed from all analyses due to errors during the sequencing process. In addition, three *X. fastidiosa* subsp. *pauca* whole genome assemblies were obtained from NCBI: COF0407 (XFAS006-SEQ-1-ASM-1, https://www.ncbi.nlm.nih.gov/assembly/GCF_001549825.1/) from coffee, OLS0478 (XFAS005-SEQ-1-ASM-1, https://www.ncbi.nlm.nih.gov/assembly/GCF_001549755.1/) from oleander, and OLS0479 (XFAS004-SEQ-2-ASM-1, https://www.ncbi.nlm.nih.gov/assembly/GCF_001549735.1/) also from oleander. Overall, this resulted on a sample size of *n* = 15 for the Costa Rican population (*n* = 12 from subsp. *fastidiosa* and *n* = 3 from subsp. *pauca*).

The quality of raw paired FASTQ reads was evaluated using FastQC [34] and visualized using MultiQC [35]. Low quality reads and adapter sequences were removed from all paired raw reads using seqtk v1.2 (https://github.com/lh3/seqtk) and cutadapt v1.14 [36] respectively with default parameters. After pre-processing, isolates sequenced with Illumina were assembled de novo with SPAdes v3.13 [37, 38] using the -*careful* parameter and -k of 21, 33, 55, and 77. A hybrid assembly of Pacbio CSS and Illumina reads was also built with SPAdes v3.13 using the -*s* parameter for the other isolates. Assembled contigs were reordered using Mauve’s contig mover function [39]. Complete publicly available assemblies were used as references. Specifically, subsp*. fastidiosa* scaffolds were reordered using the Temecula1 assembly (GCA_000007245.1), while subsp*. pauca* scaffolds were reordered using the 9a5c assembly (ASM672v1). Assembled and reordered genomes were then individually annotated using the PGAP pipeline [40] after removal of contigs shorter than 400 nucleotides. In addition, published genome sequences were also individually annotated with PGAP.

A close evaluation of isolate’s XF70 assembly and annotation suggested potential contamination during sequencing. Contaminant sequences were filtered by mapping FASTQ reads against the XF72 assembly using bowtie2 v2.3.4.1 [41] without the *–unal* parameter. The XF72 sequence was chosen because it was the closest relative to XF70 on the ML trees generated from the Costa Rica dataset (see later methods). A BAM file including reads mapped in the proper pair order was created using the *-f 2 flags* in Samtools v1.8 [42]. Subsequently, the BAM file was sorted by read name using the *-n flag*. Finally, Bedtools v2.26.0 [43] was used to convert the sorted BAM file into filtered FASTQ files. These filtered files were assembled using SPAdes v3.13 as previously described.

### Pan genome analysis of *X. fastidiosa* isolates and maximum likelihood trees

The core (genes shared between 99 and 100% strains), soft-core (genes shared between 95 and 99% strains), shell (genes shared between 15 and 95% strains), and cloud (genes shared between 0 and 15% strains) genomes were individually calculated for the complete data set (*n* = 261) and for the Costa Rica data set (*n* = 15, 12 newly assembled plus 3 published genomes). Roary v3.11.2 [44] was used to create an alignment of genes shared in 99–100% of the isolates in a dataset (core gene alignment) and to calculate a presence/absence matrix of each identified gene. The core genome alignments were used to build a Maximum Likelihood (ML) tree using RAxML [45]. All trees were built using the GTRCAT substitution model. Tree topology and branch support were assessed using 1000 bootstrap replicates.

Within the Costa Rica dataset, Roary’s presence/absence matrix was used to calculate variations on the core genome size on each node of the ML tree. In addition, the number of synapomorphies (genes shared by all isolates descended from that node and absent from any other isolates on the tree) was also quantified. These numbers were visualized using a cladogram of the Costa Rica isolates. In addition, the transposed presence/absence matrix was used to calculate the stochastic probability of gene gain/loss with the GLOMME web server [46], using default parameters. Genes within the softcore, shell, and cloud genome were categorized based on Clusters of Orthologous Groups (COG) and divided in four main functional categories: ‘Metabolism’, ‘Information storage and processing’, ‘Cellular processes and signaling’, and ‘Uncharacterized’. Genes without a defined COG category, but with a UniprotKB ID number were mapped to their corresponding COG using the KEGG Pathway Database. Genes without defined COG or UniprotKB IDs (e.g. hypothetical proteins) were assigned to the ‘Uncharacterized’ category. A heatmap was used to visualize variations in gene presence/absence for each of the four main functional categories. The individual heatmaps were built using the ‘gplots’ R package. In addition, the genetic gain/loss patterns of known virulence genes [47] was also assessed.

### Detection of recombinant sequences within the Costa Rica data set

FastGEAR [48] was used with default parameters to identify lineage-specific recombinant segments (ancestral) and strain-specific recombinant segments (recent) in the core genome alignment of the Costa Rican dataset. Non-recombinant ML trees were built after removing recombinant segments of the alignment using an *in-house* python script. Changes in tree topology and branch support between the ‘core genome’ ML trees and the ‘core genome minus recombinant segments’ ML trees were assessed. The size and location of recombinant segments between two isolates was mapped across the length of the alignment using the R package ‘circlize’ [49]. In addition, donor and recipient recombinant regions were visualized using fastGEAR’s plotRecombinations script. The number of recombination events in which a pair of isolates acted as a donor/recipient was visualized in a heatmap built with the R package ‘gplots’. The patterns of ancestral and recent recombination events between subsp. *pauca* isolates from Brazil were also calculated and compared to those observed within the Costa Rica population.

In addition to the recombination events detected between available isolates, fastGEAR also found recent recombination events involving an ‘unknown’ lineage. To evaluate the relation of this lineage with other Costa Rica isolates, each recombinant segment involving the ‘unknown’ lineage was extracted from the core genome alignment using an *in-house* python script. Individual ML trees were built for each recombinant segment using RAxML, with the GTRCAT substitution model and 1000 bootstrap replicates. Subsp*. pauca* isolates were used as the ML tree root. Trees where subsp*. pauca* isolates did not form a monophyletic clade (*n* = 10) were removed from visualizations with the R package ‘phytools’ [49]. Another *in-house* python script was used to find the ‘unknown’ recombinant segments on the core alignment of the larger dataset, which included subsp. *fastidiosa* and subsp. *pauca* isolates from diverse geographical regions (*n* = 261), and subsequently build individual ML trees as previously described.

An *in-house* python script was used to find genes contained entirely within ancestral and/or recent recombinant segments. Recombinant genes were identified using the newly annotated XF1090 genome as a model for subsp*. fastidiosa* from Costa Rica and the published COF0407 genome (XFAS006-SEQ-1-ASM-1) as a model for subsp*. pauca* from Costa Rica. The presence of functional annotation clusters that were overrepresented (enriched) within recombinant genes for each subspecies was calculated using the Functional Classification Tool included in the Database for Annotation, Visualization, and Integrated Discovery (DAVID v6.8) [50]. DAVID was used to identify and group genes with similar annotated functionality. Functional enrichment analyses were performed using all identified UniprotKB IDs obtained for XF1090 and COF0407 as a background of subsp*. fastidiosa* and subsp*. pauca* from Costa Rica, respectively. A variable number of annotation clusters were generated based on the grouped functional categories identified. Clusters were organized from those most overrepresented or with higher Enrichment Scores (ESs) (Annotation Cluster 1) to those least overrepresented or with lower ESs.

### Genetic diversity and population genetic sweeps

Global measures of genetic diversity were estimated for each subsp*. fastidiosa* population (Spain, Taiwan, Southeastern US, California, and Costa Rica) and each subsp*. pauca* population (Costa Rica, Brazil, and Italy). Genetic diversity was estimated by computing haplotype diversity (H), nucleotide diversity (π), and Watterson’s estimator (θ), within and between populations. All estimates were calculated using the entire core genome alignment for each subspecies and a second time following removal of segment with recombinant signals from each core alignment. Briefly, nucleotide diversity (π) measures the average number of nucleotide differences per site in pairwise comparisons among DNA sequences. Haplotype diversity (H), also known as gene diversity, measures the probability that two randomly sampled alleles are different. The Watterson estimator measures population mutation rate [51]. The global measures of genetic diversity were calculated for each population on individual subsp*. fastidiosa* and subsp*. pauca* core genome alignments using the R package ‘PopGenome’ [52].

In addition, the genetic diversity statistics: Tajima’s D [53] was estimated for each subsp*. fastidiosa* and subsp*. pauca* population. Given the low sample size, the statistics could not be confidently calculated on the subsp*. fastidiosa* isolates from Spain (*n* = 3) and Taiwan (*n* = 2), or in subsp*. pauca* isolates from Costa Rica (*n* = 3). Briefly, negative Tajima’s D values indicate a lower amount of polymorphism in a population than expected under neutrality. Hence, negative values can be caused by a selective sweep or a recent species introduction. On the other hand, positive values indicate a higher amount of polymorphism than expected under neutrality. Hence, positive values suggest the existence of multiple alleles in a population maintained by balancing selection or a recent population contraction. The diversity statistics were calculated for each population on individual subsp*. fastidiosa* and subsp*. pauca* core genome alignments using the R package ‘PopGenome’. Additionally, Tajima’s D estimates were calculated across the length of the core genome alignment using a sliding window of 500 nucleotide size with the R package ‘PopGenome’. Finally, in order to establish the overall effect that recombination has on *X. fastidiosa* diversity within a population (e.g. as a homogenizing and/or diversifying force), the overall Tajima’s D calculations for each population were repeated after removing the recombinant segments detected by fastGEAR. Also, the number of substitutions introduced by recombination vs. random point mutation (r/m) [54] was estimated for subsp. *fastidiosa*’s and subsp. *pauca*’s core gene alignment using ClonalFrameML [55].

Signatures of linkage disequilibrium (LD) were used to estimate the strength and location of selective sweeps within each population. In addition, the prevalence of LD signatures in different protein functional classes was also evaluated. The Rozas’ ZZ index was used to identify LD values across the length of the core genome alignment using a bin size of 500 nucleotides. The Rozas’ ZZ index [56] is quantified by comparing the Kelly’s ZnS index (average of the squared correlation of the allelic identity between two loci over all pairwise comparisons [57]) and the Rozas’s ZA index (average of the squared correlation of the allelic identity between two loci over adjacent pairwise comparisons [56]). Positive values indicate that two alleles occur together on the same haplotype more often than expected by chance, and negative values indicate that alleles occur together on the same haplotype less often than expected by chance. Index values were mapped against the location of genes within the core genome alignment. Briefly, Rozas ZZ index values were assigned to the corresponding core genome gene found within the region. In the case of genes located in multiple 500 nucleotide bins, an average of the Rozas ZZ index for those bins was obtained and subsequently assigned to the gene. Genes were categorized based on their COG and divided into five main functional categories: ‘Metabolism’, ‘Information storage and processing’, ‘Cellular processes and signaling’, ‘Uncharacterized’, and ‘Multiple’. Genes without a COG but with a UniprotKB ID number were assigned a COG using the KEGG Pathway Database. Genes without COG or UniprotKB IDs were assigned to the ‘Uncharacterized’ category. Genes with COG from multiple categories were assigned to the group ‘Multiple’. A box plot was used to evaluate the relationship between LD estimates and gene function. All LD analyses were performed using the R package ‘PopGenome’.

### Grapevine inoculation with Costa Rican *X. fastidiosa* subsp. *fastidiosa* isolates

*X. fastidiosa* mechanical inoculation assays were performed on *Vitis labrusca* grapevines, in green house conditions. Suspensions of 13 strains were prepared in Phosphate Saline Buffer (PBS) from 7-day old colonies grown on BCYE solid medium. Bacterial suspensions were prepared and homogenized to an optical density of 0.2 at 600 nm (estimate of 10^8^ to 10^9^ UFC/mL) and confirmed by colony plate technique. A 10 μL drop of the suspension was placed on a young stem of the plant, and the tissue was pricked through the drop with an entomological pin. Three sites per plant were inoculated. Three rounds of inoculation were performed (two weeks a part) for each set of plants. Each isolate was inoculated into three grape plants. We note that this inoculation procedure was expected to maximize chances of infection. Mock inoculations were done with PBS only in four control plants. Plants were monitored through a period of 6 months for the presence of symptoms. At 2- and 6-months, mature leaves near the inoculation site were collected and tested for the presence of the bacteria using culture methods [58], and indirect immunofluorescence [32]. For molecular detection, DNA was extracted from petioles using DNEASY Plant mini kit (QIAGEN), and tested using Real Time PCR (RT-PCR) [59] and Loop-Mediated Isothermal Amplification (LAMP) [60]. Unfortunately, *V. labrusca* plants naturally infected with *X. fastidiosa* were not recovered and local *X. fastidiosa* infection in grapevines could not be assessed (i.e. positive controls for the inoculation experiments). However, previous reports show that *X. fastidiosa* strains (ST18) may infect and produce PD symptoms in *V. vinifera* in Costa Rica [61] and that local infection of *X. fastidiosa* in *V. labrusca* occurs naturally [62]. In other words, while not recovered in this study, local infection of *V. labrusca* with native *X. fastidiosa* strains is likely to occur in Costa Rica.

## Results

### Gene gain/loss events are prevalent within both Costa Rican *X. fastidiosa* subspecies

**Fig. 1:**
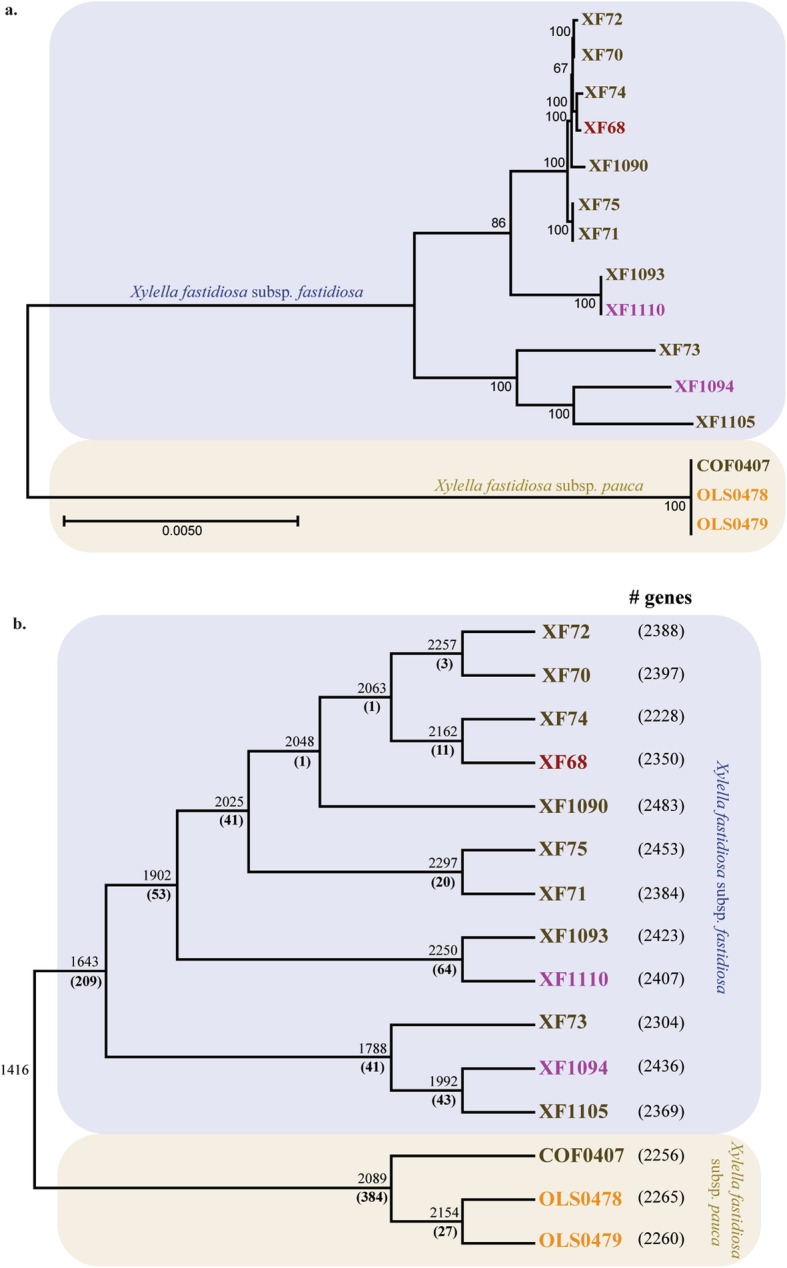
Number of shared genes among *Xylella fastidiosa* isolates. **a.** ML phylogenetic tree shows the relationship between isolates *X. fastidiosa* subsp*. fastidiosa* (blue) and subsp*. pauca* (yellow) from Costa Rica plants. Isolates names are colored based in the plant in which they were isolated: *Vinca* spp. (purple), *Psidium* spp. (red), *Coffea* spp. (brown), and *Nerium oleander* (orange); **b.** A cladogram shows the core genome (up) and the number of unique at each node of the phylogeny (down, bolded). Numbers next to isolate name indicate number of genes in the genome

A total of 4816 genes were identified in the Costa Rica dataset (12 strains for subsp. *fastidiosa* and 3 for subsp. *pauca*), with 1416 genes forming the core genome (Table 2). Isolates from subsp*. fastidiosa* and subsp*. pauca* formed two well-supported clades (Fig. 1a). A total of 1643 genes were shared only by subsp*. fastidiosa* isolates, while 2089 genes were shared uniquely among subsp*. pauca* isolates (Fig. 1b). Within the twelve subsp*. fastidiosa* isolates from Costa Rica, variations in core genome size between a node and its immediate descendant (eleven subsp. *fastidiosa* exclusive nodes) ranged from 15 to 348 genes. A difference of 65 genes was observed in the core genome size between the only two subsp. *pauca* exclusive nodes (Fig. 1b). No clear phylogenetic relation was observed between isolates infecting different plant host species. Likewise, the number of strain-specific genes was similar regardless of the host-plant species.

**Table 2:** Number of genes in the core, soft-core, shell, and cloud genomes of *X. fastidiosa* subsp. *fastidiosa* and *X. fastidiosa* subsp. *pauca* isolates included in this study, and subsp. *fastidiosa* and subsp. *pauca* isolates originating from Costa Rica. The values reported by Vanhove et al. [9] and Vanhove et al. [8] are also included

Subspecies	Core	Soft-core	Shell	Cloud
***This study***				
*X. fastidiosa* subsp. *pauca* (*N* = 101)	514	1189	860	6360
*X. fastidiosa* subsp. *fastidiosa* (*N* = 167)	1506	248	875	5246
***This study, Costa Rica (N = 15)***	1416	0	2090	1289
*X. fastidiosa* subsp. *pauca* (*N* = 3)	2089	78	107	
*X. fastidiosa* subsp. *fastidiosa* (*N* = 12)	1643	211	688	1094
***Vanhove*et al.*2019***				
*X. fastidiosa* subsp. *pauca* (*N* = 20)	1516	143	2096	1123
*X. fastidiosa* subsp. *fastidiosa* (*N* = 25)	1282	460	867	790
***Vanhove*et al.*2020***				
*X. fastidiosa* subsp. *fastidiosa* (*N* = 120)	1073	816	756	1938

The number of genes unique to each node varied between 1 to 209 among subsp*. fastidiosa* isolates and between 27 to 384 in subsp*. pauca* isolates (Fig. 1b). Even among more recently divergent sequences it was possible to observe synapomorphies. While most gene gain/loss events occur at the subspecies split, genetic gain/loss is actively occurring within each subspecies in Costa Rica. Patterns of gene gain/loss varied widely within each subspecies, with isolates from subsp*. fastidiosa* having frequent gain/loss events, particularly on the ‘Information storage and processing’ and ‘Cellular processing and signaling’ functional classes (Supplementary figure 1a-d). Isolates XF73, XF1094, and XF1105 had noticeable gene losses in the ‘Metabolism’ and ‘Cellular processes and signaling’ classes compared to other subsp*. fastidiosa* isolates. Moreover, the probability of gain/loss events for the entire pan-genome was also highest on these isolates compared to members of the same subspecies (Supplementary figure 2). In the case of known virulence genes, the largest number of gain/loss events was observed on fimbrial proteins (Supplementary Table 2). Certain fimbrial proteins seem to have experience several gain/loss events in both subspecies analyzed (e.g. *pilA_1*, *pilA_2*). Alternatively, other virulence genes (e.g. *cspA*, *gumD*, *gumH*, *pglA*, *phoP*, *rpfG*, *tolC*, and *xpsE*) are conserved in both subspecies.

### Complex recombination patterns are observed within Costa Rica *X. fastidiosa* isolates

**Fig. 2:**
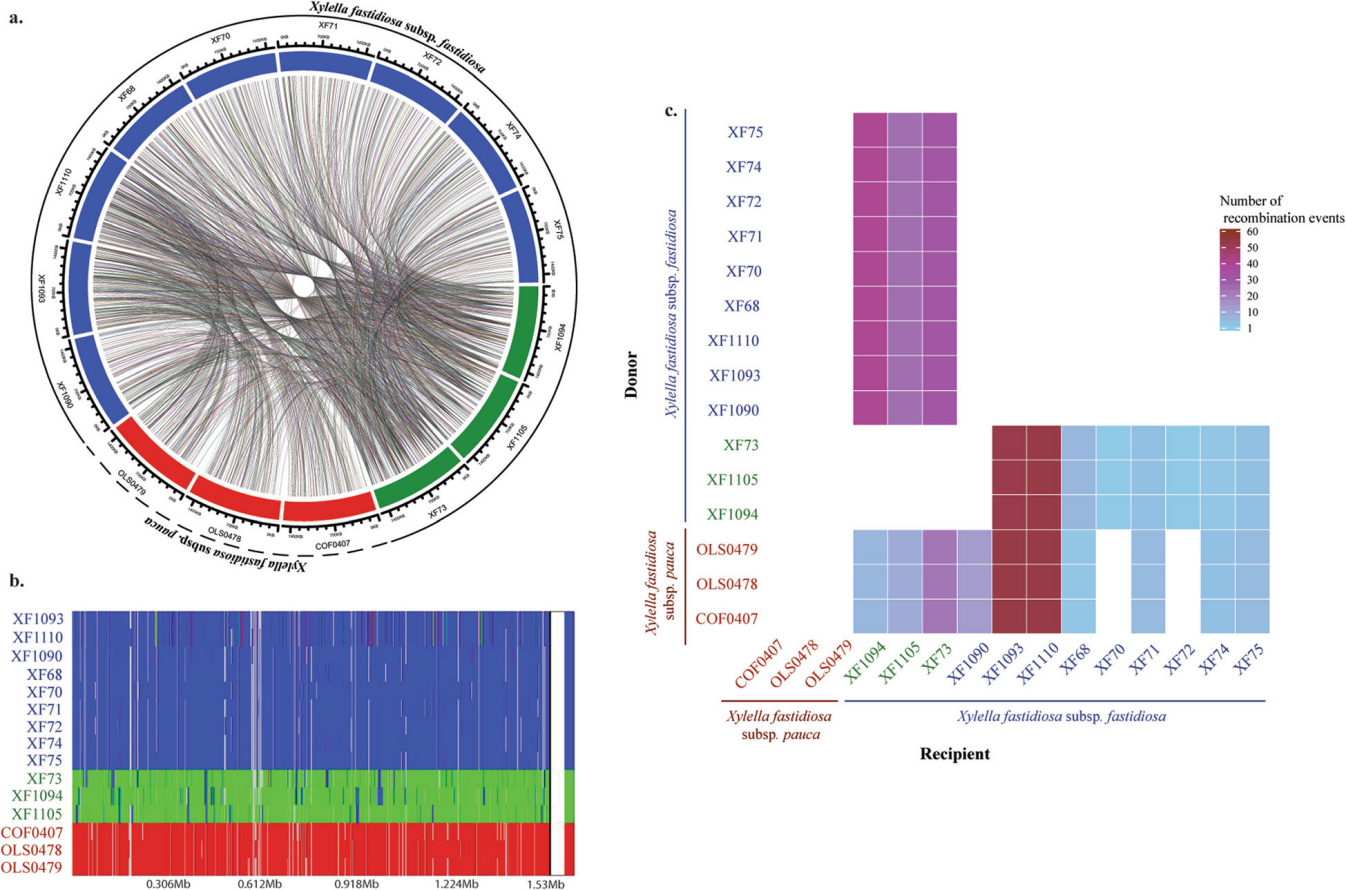
Recent recombination of two *X. fastidiosa* subspecies in Costa Rica. Colors indicate phylogenetically distinct *X. fastidiosa* lineages: *X. fastidiosa* subsp*. pauca* (red), *X. fastidiosa* subsp*. fastidiosa* (group 1, blue), and *X. fastidiosa* subsp*. fastidiosa* (group 2, green). **a.** Circular plot of strain-specific recombination events. Each line represents a recombinant event, with the width and placement of the line indicating recombinant segment size and its alignment position, respectively; **b.** FastGEAR recombination plot showing donor/recipient sequences and the position of the recombinant event in the alignment; **c.** Heatmap showing the number of donor/recipient interactions among strains

**Fig. 3:**
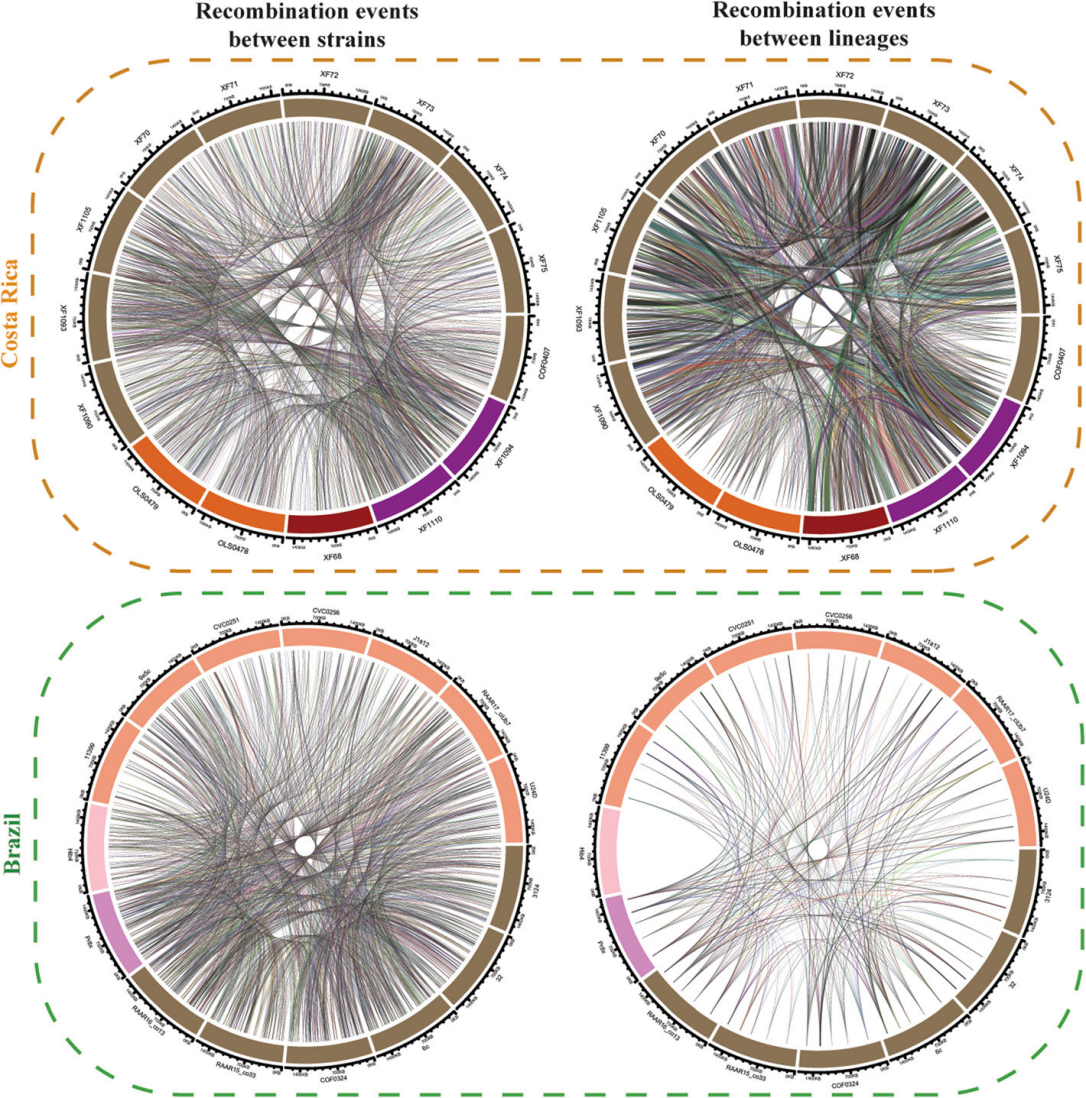
Brazil and Costa Rica lineage- and strain-specific recombination circular plots. Orange box includes isolates representing the two monophyletic clades/subspecies found within Costa Rica: *X. fastidiosa* subsp. *fastidiosa* (*Psidium* spp., *Vinca* spp. and *Coffea* spp.) and *X. fastidiosa* subsp. *pauca* (*Nerium oleander* and *Coffea* spp.). Green box includes isolates representing the phylogenetic diversity of *X. fastidiosa* subsp. *pauca* found in Brazil: the monophyletic citrus clade, the paraphyletic coffee clade, and the individual *Hibiscus* spp. and *Prunus domestica* isolates. Colors indicate isolates obtained from different host plant species. Costa Rica: *Psidium* spp. (red), *Vinca* spp. (purple), *Coffea* spp. (brown), *Nerium oleander* (orange). Brazil: *Coffea* spp. (brown), *Citrus* spp. (pale orange), *Hibiscus* spp. (pale pink), and *Prunus domestica* (dark pink). Circular plot shows recombination between strains and recombination between lineages. Each line represents a recombinant event, with the width and placement of the line indicating recombinant segment size and alignment position

The core genome alignment for the Costa Rica dataset was used to evaluate the frequency, size, and location of recombination events. Isolates were classified both based on phylogenetic relationships (Fig. 2a and Supplementary figure 3a) and plant host species (Fig. 3). Few ancestral recombination events were observed between subsp*. fastidiosa* and subsp*. pauca*. In all ancestral events observed, subsp*. fastidiosa* isolates acted as donors to subsp*. pauca* (Supplementary figure 3c). The direction of donor/recipient events flipped on recent recombination events, with subsp*. pauca* acting as a frequent donor to subsp*. fastidiosa* but never as a recipient (Fig. 2c).

In addition, the patterns of recombination were also markedly different in each Costa Rican subspecies. While ancestral and recent recombination were pervasive within subsp*. fastidiosa* isolates, no recent recombination events were observed within subsp*. pauca* isolates (Fig. 2a and Supplementary figure 3a). Within subsp*. fastidiosa*, recent and ancestral recombinant events were observed mainly between two groups of isolates. The first group included isolates XF68, XF70, XF71, XF72, XF74, XF75, XF1090, XF1093, and XF1110 (Fig. 2 and Supplementary figure 3, shown in blue); and the second group included isolates XF73, XF1094, and XF1105 (Fig. 2 and Supplementary figure 3, shown in green). Among ancestral recombinant events (Supplementary figure 3b and 3c), isolates of the first group were donors to the second group. However, both subsp*. fastidiosa* groups acted as recipient/donors during recent recombination events (Fig. 2b and c). Individual subsp*. fastidiosa* sequences participated in recombination events with variable frequency (Fig. 2c). Strains XF73, XF1094, and XF1105 were frequent donors to subsp*. fastidiosa* strains from group 1 (Fig. 2b and c), while sequences XF1093 and XF1110 were frequent recipients for both subsp*. fastidiosa* strains from group 1 and subsp*. pauca*. Overall, no specific functions were enriched in ancestral or recent recombinant genes when compared to all assigned functions on the genome (Supplementary Table 3).

Seventy-three recent recombination events out of 480 detected events involved an ‘unknown’ lineage acting as a donor sequence to isolates XF1093, XF1110, XF1094, XF1105, and XF73. The placement of each ‘unknown’ recombinant segment varied among individually built ML trees (Supplementary figure 4). Overall, in relation to other strains in Costa Rica ‘unknown’ sequences were either ancestral to other subsp. *fastidiosa* isolates (shown in red) or part of a recently divergent group (shown in purple). These results are indicative of at least two ‘unknown’ subsp. *fastidiosa* lineages circulating within Costa Rica. Furthermore, 71 of these 73 events were also found in the core genome of the complete dataset (*N* = 261) (Supplementary figure 5). These segments had three distinct phylogenetic placements: clustered within subsp. *fastidiosa* (shown in purple), clustered within subsp. *pauca* (shown in green), and ancestral to subsp. *fastidiosa* and/or subsp. *pauca* (shown in red). For one segment ancestral to subsp. *fastidiosa*, BLAST showed a 78% sequence identity and an e-value of 2e^− 07^ to *Glaesserella parasuis*, a Gram-negative bacteria found in porcine upper respiratory tracts.

Finally, a comparison of the number and nature of the recombination events within Brazil and Costa Rica (native regions to subsp. *pauca* and subsp. *fastidiosa*, respectively) showed that ancestral recombination events were less frequent in subsp*. pauca* isolates from Brazil compared to subsp*. fastidiosa* isolates from Costa Rica (Fig. 3). In contrast, the frequency of recent recombination events was similar in both populations.

### Introduction events have a significant effect on genetic diversity

**Fig. 4:**
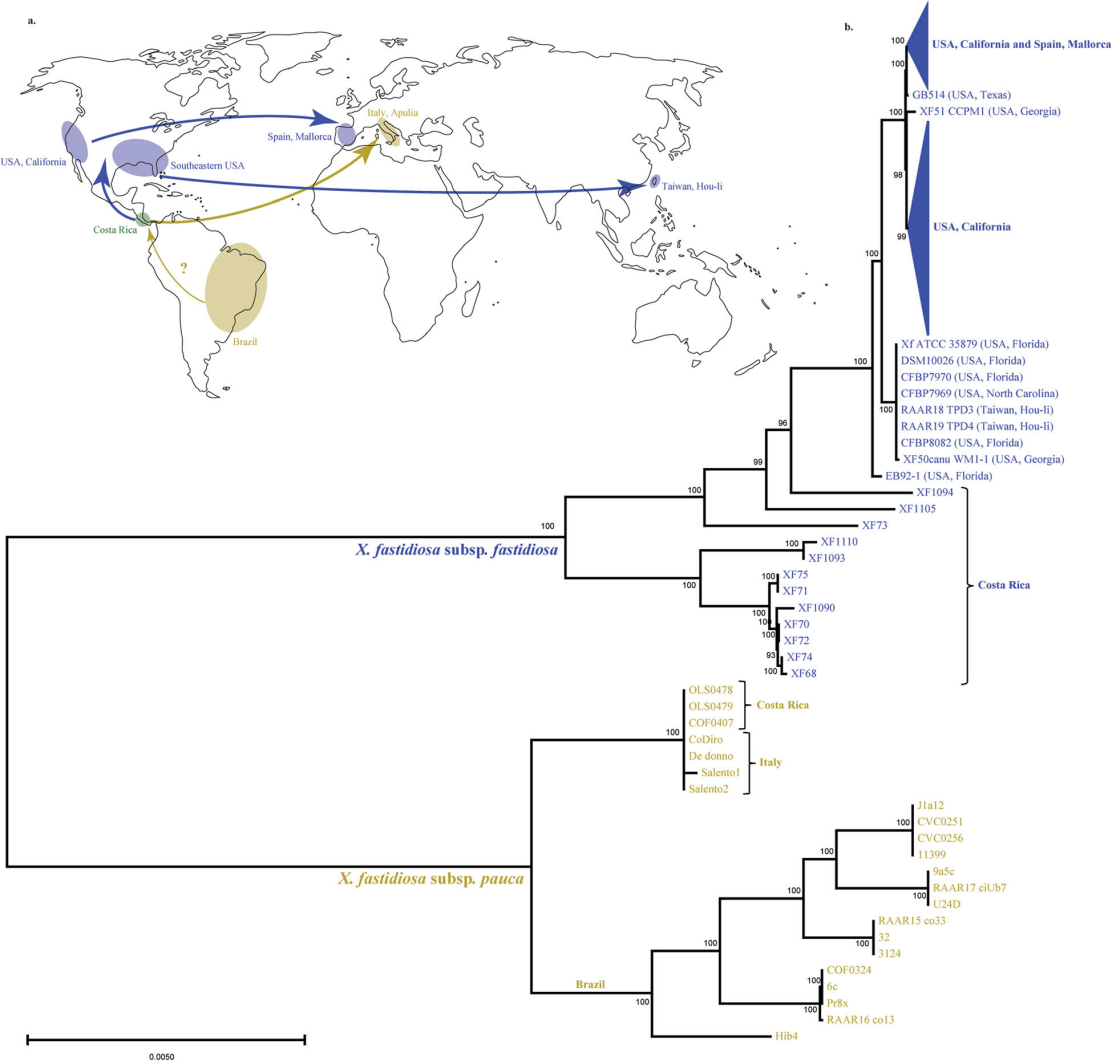
ML tree showing the relationship between worldwide *X. fastidiosa* subsp*. fastidiosa* and subsp*. pauca* isolates. **a.** Distribution map of *X. fastidiosa* subsp. *fastidiosa* (blue) and subsp. *pauca* (yellow) populations. Regions where both species coexist are marked in green. Arrows indicate source and destination populations for each introduction. Map was downloaded from Wikimedia Commons (https://commons.wikimedia.org/wiki/Maps_of_the_world#/media/File:Simplified_blank_world_map_without_Antartica_(no_borders).svg). **b.** ML tree shows the relationship of worldwide *X. fastidiosa* subsp. *fastidiosa* and subsp. *pauca* populations. Bootstrap values show support for each tree node

Both the genetic and haplotype diversity were higher within native populations of subsp. *fastidiosa* and subsp. *pauca* compared to their respective introduced populations. In specific, within subsp*. fastidiosa*, both π and H showed higher levels of diversity in Costa Rica compared to California (Table 3). These trends were maintained even when recombinant segments and low-quality sequences were removed from the core gene alignment. In the case of subsp. *pauca*, both genetic and haplotype diversity were higher in Brazil isolates compared to both Costa Rica and Italy (Table 3). Italian isolates were more genetically diverse than Costa Rican ones, despite Central America acting as a source for the Italian subsp*. pauca* outbreaks (Fig. 4). The low diversity of subsp*. pauca* from Costa Rica was likely affected by the low sample size (*n* = 3). Multiple attempts either to isolate or to maintain subsp. *pauca* in in vitro conditions have been unsuccessful (Carlos Chacón-Díaz, *personal communication*). Immunofluorescence assays have confirmed the presence of abundant bacteria in plants (~ 10 per microscopic field (10^6^ bact/g tissue)), suggesting that these issues are not due to low bacterial concentration in plants (Carlos Chacón-Díaz, *personal observation*). Therefore, these isolates may require unique in vitro conditions to support growth. Overall, while is not possible to evaluate a larger sample of subsp. *pauca* genomes at present, laboratory assays confirm its presence in Costa Rica and its virulence to local flora [63, 64].

**Table 3:** Genetic diversity (π), haplotype diversity (H), Watterson (θ), and Tajima’s D of *X. fastidiosa* subsp. *fastidiosa* (California US and Costa Rica) and *X. fastidiosa* subsp. *pauca* (Italy, Costa Rica, and Brazil) with and without recombinant regions

Population	With recombination						Without recombination					
	SNPs	Core (nt)	Nucleotide diversity (π)	Haplotype diversity (H)	Watterson’s estimator (θ)	Tajima’s D	SNPs	Core (nt)	Nucleotide diversity (π)	Haplotype diversity (H)	Watterson’s estimator (θ)	Tajima’s D
*X. fastidiosa subsp. fastidiosa*												
California (141)	452	1,401,004	2.81x10e^−05^	6.87x10e^−07^	5.85x10e^−05^	−1.712	334	957,139	4.59x10e^−05^	9.87x10e^−07^	6.35x10e^−05^	−0.912
Costa Rica (12)	14,573		3.18x10e^−03^	7.14x10e^−07^	3.44x10e^−03^	−0.37	10,024		3.19x10e^− 03^	1.04x10e^− 06^	3.47x10e^− 03^	−0.385
South East US (9)	1206		2.97x10e^−04^	7.14x10e^−07^	3.17x10e^− 03^	−0.33	942		3.32x10e^−04^	1.04x10e^− 06^	3.62x10e^− 04^	−0.441
Spain (3)	3		1.43x10e^−06^	7.14x10e^−07^	1.42x10e^− 06^	*	2		1.39x10e^−06^	1.04x10e^−06^	3.39x10e^− 06^	*
Taiwan (2)	6		4.28x10e^−06^	7.14x10e^−07^	4.28x10e^− 06^	*	4		4.18x10e^−06^	1.04x10e^−06^	4.18x10e^− 06^	*
*X. fastidiosa subsp. pauca*												
Italy (78)	16	510,568	1.12x10e^−06^	5.01x10e^− 07^	6.45x10e^− 06^	−3.022	9	261,720	1.04x10e^− 06^	4.09x10e^− 07^	7.07x10e^− 06^	−2.234
Brazil (15)	4291		3.02x10e^−03^	1.90x10e^−06^	2.58x10e^− 03^	0.592	2158		2.95x10e^−03^	3.53x10e^−06^	2.54x10e^−03^	0.731
Costa Rica (3)	3		3.92x10e^−06^	1.96x10e^− 06^	3.92x10e^− 06^	*	2		5.09x10e^−06^	3.82x10e^− 06^	5.09x10e^− 06^	*

* Costa Rica subsp. *pauca* isolates (*N* = 3) were not included

* Spain subsp. *fastidiosa* isolates (*N* = 3) were not included

* Taiwan subsp. *fastidiosa* isolates (*N* = 2) were not included

The diversity statistic Tajima’s D and the mutation rate estimate Watterson’s θ were used to quantify the effects of population history on genetic diversity and estimate population’s mutation rate; respectively (Table 3). In addition, r/m was used to measure the number of substitutions introduced by recombination relative to point mutations within core gene alignments. All diversity statistics were negative for subsp*. pauca* isolates from Italy; in contrast all values were positive for the population from Brazil. The magnitude of these diversity statistics varied after recombinant segments were removed; however, the trends (negative or positive) remained unchanged. In addition, native populations of subsp. *pauca* shower higher Watterson’s θ estimates (Table 3). On the other hand, *X. fastidiosa* subsp*. fastidiosa* populations in California, Southeastern US, and Costa Rica had negative Tajima’s D estimates, with the magnitude of these values being larger in California compared to Southeastern US and Costa Rica. These trends in these estimates remained unchanged once recombinant segments were removed. Furthermore, Watterson’s θ estimates were comparable between the Costa Rica and Southeastern US populations, but higher in California, Spain, and Taiwan. Finally, r/m rates were higher in subsp. *pauca* core alignment compared to subsp. *fastidiosa*.

Both Tajima’s D and LD (measured using the Rozas’ ZZ) estimates varied across the length of the core genome alignment (Supplementary figure 6 and 7). Those populations with small sample size (subsp. *pauca* in Costa Rica; and subsp. *fastidiosa* in Spain and Taiwan) were not evaluated. In the case of subsp. *pauca*, Tajima’s D values in the native Brazil population were highly variable across the length of the core genome, thought they largely remained positive. In contrast, only a few negative peaks were observed in the Italian population. In the case of subsp*. fastidiosa*, Tajima’s D estimates also varied across the length of the genome, particularly in the Costa Rican population. Both introduced populations (California and Southeastern US) showed sporadic positive and negative peaks in different genomic regions (Supplementary figure 6).

In the case of LD detection, the Rozas’ ZZ index was selected since it has been known to perform best in populations with larger sample size and even in the case of sudden population expansions or contractions [65]. In both native populations (subsp*. pauca* in Brazil and subsp*. fastidiosa* in Costa Rica), the Rozas’ ZZ index varied largely across the length of the core genome. This shows that co-occurrence of neighboring alleles (LD) was not limited to specific genome regions within these populations. In the case of introduced populations, few (subsp*. fastidiosa* in California and Southeastern US) or no (subsp. *pauca* in Italy) Rozas’ ZZ peaks were observed (Supplementary figure 7). This shows that allele co-occurrence (LD) is more localized in introduced populations than in native ones.

Haplotype-based methods have a high-performance detecting LD within a population; nonetheless, their power is significantly affected by frequent recombination [65]. When the distribution of LD peaks was compared with the location of recent recombination events no clear relation between both measures was observed (Supplementary figure 7). Furthermore, no clear difference was observed in the median Rozas’ ZZ index between different functional categories on either population (Supplementary figure 8).

### *X. fastidiosa* subsp. *fastidiosa* strains from Costa Rica do not cause disease in *V. labrusca* grapevines

*V. labrusca* inoculations were performed using both coffee- (XF69, XF70, and XF73) and periwinkle- infecting (XF1094 and XF1110) strains of subsp. *fastidiosa*. After 2 to 6 weeks post-inoculation none of the plants showed symptoms associated with *X. fastidiosa* infection. Likewise, immunofluorescence assays were mostly negative. Furthermore, isolation was only possible from two plants (infected with XF73) 6 weeks post-inoculation. The number of positive infections (RT-PCR (10/15) and LAMP (8/15)) detected 2 months post-inoculation was markedly reduced 6 months post-inoculation (RT-PCR (5/15) or LAMP (2/15)) indicating that infections are not sustained. Overall, our results show that the tested native subsp. *fastidiosa* from Costa Rica is not capable of successfully infecting *V. labrusca* (Supplementary Table 4).

## Discussion

### Change in core and accessory genome size show a highly malleable *X. fastidiosa* genome

In bacteria, the size of the pan genome and its relationship with the core genome are largely influenced by the environment [66]. For instance, changes in the environment can result in gene loss [66] due to the relaxation of purifying selection, and can also reduce fitness costs [67]. Likewise, gene gain has been associated with environmental adaptation [68]. The patterns observed in free-living bacteria are likely also present in pathogens such as *X. fastidiosa*, where environmental changes are analogue to host switch events. Moreover, host switches originate both from introductions to new locations and/or by infections of novel/naïve plant species within the original location, as well as the requirement for vector transmission.

The core genome shared by both subspecies within Costa Rica was roughly 86% of the size of the core genome of subsp. *fastidiosa*’s core genome and 67% of the size of subsp. *pauca*’s core genome. Moreover, variations on the core and accessory genome size were more prominent within Costa Rican subsp. *fastidiosa* compared to subsp. *pauca*. Previous studies have shown than gain/loss events are less frequent in introduced subsp. *fastidiosa* isolates from the continental United States compared to subsp. *pauca* isolates from Brazil (native), Costa Rica (introduced), and Italy (introduced) [9]. This could suggest that gain/loss events are more frequent in older populations compared to recently introduced ones. In the case of subsp. *fastidiosa* isolates from Costa Rica, the frequent gene gain/loss events could also reflect a more diverse set of available plant hosts. Bacterial gene gain/loss events are common in diverse biotic environments with many potential host-pathogen partners [66]. Thus, the variations in the accessory and core genome size seen within subsp. *fastidiosa* are potentially the product of a long-term ecological history involving multiple available host environments.

The number of core genome genes in Costa Rican subsp. *fastidiosa*’s and subsp. *pauca*’s isolates was higher than in previous reports [9]. These differences are largely a product of the distinct datasets used on each study. Specifically, Vanhove et al. (2019) incorporated isolates from populations of diverse geographical origins, while Costa Rica represents a single geographic point. When both datasets are combined with Vanhove et al. (2020) and Sicard et al. (2020), the raw number of core and soft-core genes changed; however, the combined estimates for both categories remain roughly the same. This suggests that both subspecies have a largely stable core genome with most gain/loss events being limited to the accessory genome. It should be noted however, that estimates related to the core and accessory genome are sensitive to the algorithms used by diverse annotation software, with distinct software potentially generating conflicting results. Moreover, the quality of de novo annotations affects such analyses.

Within Costa Rica, isolates obtained from distinct plant species did not cluster within a single clade, supporting the idea that host switching within Costa Rican subsp. *fastidiosa* is not associated with small nucleotide changes within a conserved set of genes, and instead might be the product of complex gene gain/loss patterns. However, it must be pointed out that except for XF68 (isolated from guava, *Psidium* spp.), all isolates originate from plant species introduced to Costa Rica: *Coffea* spp. was introduced near the end of the eighteenth century when in gained significant commercial importance [69], while *Nerium oleander* and *Catharanthus roseus* (*Vinca* spp.) might have been introduced in multiple occasions for their use as ornamental plants in house gardens. Furthermore, the number of isolates included here is small. Therefore, including additional isolates from less explored hosts plants within this regions, if possible, would aid to confirm how gene gain/loss influences host switching. Sicard et al. (2018) proposed that *X. fastidiosa* adaptation to a given host is limited in more diverse plant communities, with natural selection favoring higher bacterial genetic diversity and the development of non-pathogenic generalist genotypes. The absence of plant host monophyly observed in the Costa Rican and Brazilian populations suggest that when potential plant host diversity is limited, host switching in native *X. fastidiosa* populations may occur via convergent evolution. Overall, the larger genetic diversity of native *X. fastidiosa* populations may facilitate host switching, while the limited genetic diversity of monocultures enables repeated colonization of the same host species and pathogen specialization.

The probabilities of gene gain/loss were highest in strains XF73, XF1094, and XF1105. These isolates formed a monophyletic group within the Costa Rica dataset and a gradient leading to the introduction to the continental US when the entire subsp. *fastidiosa* dataset was used. Among all subsp. *fastidiosa* isolates within Costa Rica, the number of gene gain/loss events was roughly similar across functional groups. This indicates that other than genes essential for survival (likely found within the core genome), genetic gain/loss was not selected by specific functional class. On the contrary, these results suggest that these events are ubiquitous and may involve multiple functional groups simultaneously. Moreover, clear trends in gene gain/loss were not observed in known virulence genes. Instead, these genes were largely conserved in all Costa Rican isolates as well as within subsp. *fastidiosa* and subsp. *pauca*. Only genes coding for fimbrial proteins (e.g. *pilA*) were repeatedly gained and lost in native populations of subsp. *fastidiosa* and subsp. *pauca*, with at least two *pilA* copies being conserved in all isolates from both subspecies. The potential significance of *pilA* gain/loss events in twitching and motility [12, 70] and its putative role in *X. fastidiosa* virulence remains to be evaluated; however, these results suggest that gene duplication might be evolutionarily important for certain virulence factors.

The monophyletic group containing XF73, XF1094, and XF1105 showed distinct gain/loss patterns compared to other subsp. *fastidiosa* isolates. Furthermore, even within the core genome, isolates XF73, XF1094, and XF1105 had pronounced sequence divergence compared to other subsp. *fastidiosa* isolates within Costa Rica. Moreover, these isolates also formed a highly recombinant cluster within subsp. *fastidiosa*. Previous studies have suggested that homologous recombination leads to homogenization of the core genome in bacteria [71]; however, this does not appear to be the case here. Instead, isolates XF73, XF1094, and XF1105 diverged from other subsp. *fastidiosa* within the core (sequence divergence) and the accessory genome (gene gain/losses). Whether high recombination rates led to high sequence divergence in XF73, XF1094, and XF1105 or ancestral sequence divergence lead to higher recombination rates within these sequences cannot be currently established.

### Homologous recombination is a major source for evolutionary change within native *X. fastidiosa* populations

Within Costa Rica, genetic exchange mediated by recombination was strain and time dependent. Ancestral recombination events were prevalent within subsp. *fastidiosa* but not within subsp. *pauca*. Moreover, subsp*. fastidiosa* acted as a donor to introduced subsp*. pauca* isolates in ancestral introgression events, but not as a recipient. The direction of donor/recipient strains within Costa Rica changed with evolutionary time. This suggests that the directionality of donor/recipient events can be time dependent, with introduced subspecies being more likely to act as donors to established *X. fastidiosa* strains as time passes. Within recent recombination events, subsp. *pauca* acted as a frequent donor to specific subsp. *fastidiosa* strains. This could imply that new alleles could be beneficial and subsequently retained by native populations. While our sample size for this region, and particularly subsp. *pauca* is small, previous studies have found evidence that introduced *X. fastidiosa* subspecies can often act as allele donors to a local population [23, 27, 28]. Previous studies have shown that genetic exchange within local and introduced *X. fastidiosa* isolates occurs in short evolutionary time and despite ecological isolation [28]. This could explain the increment in the number and complexity of recombination events seen here. Within subsp. *multiplex*, recombinant genotypes form well defined phylogenetic groups, suggesting that recombination among isolates might not be equally distributed [20]. Furthermore, recombination frequency also varies among strains [72]. Our results show that this is also the case in native populations of subsp. *fastidiosa*, with frequently recombinant isolates having a unique placement in the ML tree.

In addition, at least two ‘unknown’ recombinant lineages were found in both the Costa Rican *X. fastidiosa* population as well as in isolates from other geographical regions. The presence of unidentified lineages indicates a high diversity of recombinant segments in *X. fastidiosa* populations. Moreover, their distinct phylogenetic placements imply independent geographical origins (i.e. introduced vs. native), associated subspecies (i.e. subsp. *pauca* vs. subsp. *fastidiosa*), and evolutionary ages (i.e. ancestral vs. derived lineages).

To determine if strain-specific recombination capacities could be a trend of native populations, a similar analysis was performed on subsp. *pauca* isolates from Brazil. *X. fastidiosa* subsp. *pauca* strains infecting Brazilian coffee and citrus plants are genetically distinct [73] but have been found to recombine [23, 74]. Our in silico analyses show that genome-wide recombination occurs within native subsp. *pauca* isolates. Moreover, the frequency of recombination increases with time (ancestral vs. recent). Only ecological factors such as geographic distance (i.e. Hib4, assembly GCA_001456315.1, separated from other Brazilian strains by ~ 300 miles) seemed to limit recombination events. Therefore, our results indicate that with enough evolutionary time, genetic exchange will happen among co-occurring *X. fastidiosa* populations. Furthermore, elements of local population history, such as the outbreak’s age and the unique ecology on each region, are likely to have a strong influence in the quantity and direction of recombination events in *X. fastidiosa*.

No specific functions were enriched in ancestral or recent recombinant genes compared to all assigned functions on the genome. It has been proposed that while recombination is an important source for the development of genetic diversity and host adaptation [75], loss of fitness often leads to inter-subspecific recombination events to fail [22]. Previous studies have suggested that levels of recombination vary among subspecies and that recombination events are widespread across the *X. fastidiosa* genome, with certain genomic regions potentially acting as recombination hotspots [76]. The pervasive levels of recombination observed here suggest that within natural populations of *X. fastidiosa*, homologous recombination is a genome wide occurrence. Thus, frequently recombining genes might be, for the most part, those with lower fitness cost or benefits to natural populations. Moreover, the lack of functional association of recombination events suggests that recombinant-rich regions could be a product of physical properties of the genome and not reflect adaptive potential.

### Changes on genetic diversity and linkage disequilibrium showcase a history of *X. fastidiosa* introductions and adaptation

The current knowledge on *X. fastidiosa* indicates that at least three of the recognized subspecies are allopatric within the American continent [18, 77]. Multiple anthropogenically facilitated long-distance dispersal events have expanded the geographic ranges and scope of plant hosts affected by *X. fastidiosa* [31]. The low genetic diversity of the subsp. *fastidiosa* California population obtained via MLST has been considered as evidence for a recent introduction from Central America [11], as well as its phylogenetic placement in relation to isolates from Costa Rica. Our preliminary results support the hypothesis that subsp. *fastidiosa* was introduced to the US from Central America. At a whole genome level, the nucleotide diversity in Costa Rica was the highest of all evaluated populations (Costa Rica, 14,573 SNPs and π = 3.18x10e^− 03^; Southeastern US, 1206 SNPs and π = 2.97x10e^− 04^; California, 452 SNPs and π = 2.81x10e^− 05^; Spain, 3 SNPs and π = 1.43x10e^− 06^; and Taiwan, 6 SNPs and π = 4.28x10e^− 06^). Moreover, this trend was maintained even after removing recombinant regions (Costa Rica, 100,24 SNPs and π = 3.19 × 10^− 03^; Southeastern US, 942 SNPs and π = 3.32 × 10^− 03^; California, 334 SNPs and = 1.18x10e^− 04^; Spain, 2 SNPs and π = 1.39x10e^− 06^; and Taiwan, 4 SNPs and π = 4.18x10e^− 06^). The nucleotide diversity values obtained here are lower than those previously reported for both subsp. *fastidiosa* [9] and uniquely California [8]. In addition, the r/m estimates for subsp. *fastidiosa*’s core gene alignment (r/m = 2.074) were lower than those previously reported for this subspecies (r/m = 4.059 [9] and r/m = 6.797 [8]). These differences can be explained by the distinct groups of isolates included on each study. Mainly, the first study [9] included a smaller number of isolates from the continental United States, which could potentially result in larger nucleotide diversity as well as different recombination trends being detected. The focus in the second study [8] was to estimate local adaptation within California. Because of this, genetic diversity calculations in Vanhove et al. (2020) would consider California-specific SNPs that are likely absent from our dataset.

The diversity estimate Tajima’s D was negative in Costa Rica, Southeastern US, and California, thought the magnitude was lower in California. Given the importance of recombination in host-switching [28] and in genome adaption and plasticity [9], we attempted to estimate the impact of homologous recombination on these diversity estimates. Within the Costa Rican and Southeastern US populations, diversity estimates remained largely similar after recombinant regions were removed, suggesting that this is not the only contributing force to developing and maintaining genetic diversity. These trends could be the result of specific evolutionary forces acting on each population. For instance, Watterson estimates are similar in both populations but slightly vary once recombinant segments are removed. Without recombining regions, the Costa Rica population has a higher mutational rate estimate than Southeastern US. This could suggest that mutation rates are higher in the native subsp. *fastidiosa* population.

On the other hand, the influence of population specific ecology or even the isolate’s origin also has a role in these diversity estimates. While most California isolates were obtained from *Vitis vinifera* crops in one state; isolates form the Southeastern US encompass a larger area (Georgia, Florida, Texas, and North Carolina). Previous studies have shown evidence of local adaptation within subsp. *fastidiosa* populations in California [8]. It is possible that these trends are more pronounced in a larger region, thus leading to an overall increase in genetic diversity. In addition, isolates from the Southeastern US also originate from diverse plant host species: *Vitis* spp., *Sambucus* spp., and *Ambrosia artemisiifolia*. Plant host specialization has been described both in subsp. *multiplex* [20] and subsp. *pauca* [74]. Moreover, there is a strong association between appearance of symptoms in oleander plants from Costa Rica and the detection of clade ST53. Thus, host-specific adaptations could also contribute to the higher genetic diversity of the region.

In the California population Tajima D’s estimates remained negative after recombinant regions were removed; however, there was a clear increase in their overall magnitude. This would be consistent with the genetic diversity of recombinant regions being lower than non-recombinant ones. While inter-subspecific recombination is a major force for genomic diversification within *X. fastidiosa*, these results could suggest that it might also facilitate homogenization [71] on specific genome regions and within certain populations. Moreover, the lower diversity of recombinant regions could be further enhanced by the fixation of specific beneficial alleles due to the action of selective sweeps [78].

Similarly, the diversity trends observed on subsp*. pauca* populations were consistent with pathogen introductions. Both the low nucleotide diversity and the negative diversity statistic values support the recent introduction of subspecies *pauca* to Italy from Central America [79]. In contrast, the high nucleotide diversity and positive diversity statistics imply that South America (e.g. Brazil) is maintaining multiple alleles over time, which matches the proposition that this is the native population of subsp*. pauca*. Furthermore, r/m estimates (r/m = 3.365) closely resemble those previously reported (r/m = 3.727) [9]. A strong relation between recombination rates and increased genetic diversity has been reported in Brazil [28], this might also be the case in other native populations of this bacterium. This suggests that homologous recombination might not be the only factor determining genetic diversity in native subsp. *pauca* and that other factors, such as mutation rate, might also be important.

The positive Rozas’ ZZ values found across the length the core genome alignment suggests that variable LD signatures are present in multiple genes in both native populations. Moreover, no clear relation between LD and homologous recombination was observed, suggesting that additional evolutionary forces might influence allele distribution. In addition, absence of a clear relation between the median Rozas’ ZZ index and gene functional categories indicates that no individual category is under stronger linkage than others. Furthermore, no clear relationship was observed even when the distribution of Rozas’ ZZ values as a function of individual COG codes was evaluated via an ANOVA (F = 0.876, *p*-value = 0.849 for subsp. *fastidiosa*; and F = 0.949, *p*-value = 0.764 for subsp. *pauca*). LD signatures can be the product of multiple evolutionary forces, including: positive selection, recombination, population bottlenecks, and/or genetic drift [80]. Natural selection alone is unlikely to results in the widespread evidence of LD patterns observed here, thus multiple evolutionary forces must be acting simultaneously within native *X. fastidiosa* populations. Studies on *Staphylococcus aureus* describe a dramatic decrease of LD in distances larger than 10 kb due to homologous recombination [81]. Alternatively, homologous recombination can disrupt linkage between sites under different selective constraints, increasing natural selection’s efficiency [82, 83]. Thus, it is possible that the LD patterns observed within Costa Rica and Brazil are a product of complex interactions between natural selection and recombination. On the other hand, both California and the Southeastern US populations showed a few positive peaks scattered across the length of the core genome alignment. This suggests that LD is found only within a few core genes in each population, a pattern indicative of haplotype loss due to the recent population bottleneck and subsequent expansion.

### *X. fastidiosa* subsp. *fastidiosa* strains from Costa Rica do not cause disease in *V. labrusca* grapevines

*X. fastidiosa* subsp. *fastidiosa* strains from Costa Rica were not capable to cause disease symptoms in *V. labrusca*. Infection was detected in a small number of plants via RT-PCR and LAMP; nonetheless, the number of positively detected plants decreased with time (from 2- to 6- months post inoculation) and the identity of those plants was inconsistent. The strains used for inoculation: a) originated from both coffee- or *Vinca*-infecting plants, b) clustered in two distinct phylogenetic clades, and c) included both more recombinant (XF73 and XF1094) and less recombinant (XF70 and XF1110) strains. Overall, these results suggest that the capacity to successfully infect grapevines and cause Pierce's Disease is not ancestral to subsp. *fastidiosa*. Previous studies [27, 74] have shown that host switches on *X. fastidiosa* occur via multiple evolutionary mechanism and ecological circumstances. Thus, it is possible that the host switch to grapevines resulted from both locally adaptive (i.e. positive selection to a monoculture) and non-adaptive (i.e. founder effect) evolutionary forces acting in conjunction.

### *X. fastidiosa* subsp. *fastidiosa* from Costa Rica is ancestral to other geographic locations

The phylogenetic relations among subsp*. fastidiosa* and subsp*. pauca* isolates from diverse geographic locations, shows two clearly defined clades marking the separation of subsp*. fastidiosa* and subsp*. pauca*. The distinct branch length and topology within every cluster inside each clade showcases unique evolutionary patterns that are both location and subspecies dependent. Costa Rican isolates are found at the base of the subsp*. fastidiosa* clade, with all other locations deriving from them. Moreover, branch length between Costa Rican isolates show substantial amounts of evolutionary change comparable to that observed in the native subsp. *pauca* isolates from Brazil. This supports the hypothesis of Central America as the geographic point of origin for subsp. *fastidiosa* [18]. In addition, our results further support the hypothesis that subsp*. fastidiosa* was introduced to the US from Central America which is based on extensive MLST analyses performed in both regions [11, 64]. Overall, our study shows complex gene gain/loss patterns were identified among subsp. *fastidiosa* isolates from distinct plant origins, and widespread evidence of intra-subspecific recombination was detected among subsp. *fastidiosa* isolates. In addition, higher genetic diversity was measured in the Costa Rican subsp. *fastidiosa* population compared to other subsp. *fastidiosa* populations, and even the Costa Rican subsp. *pauca* population. Moreover, isolates obtained from recent outbreaks in Mallorca, Spain and Hou-li, Taiwan show a close evolutionary relationship with populations in California and the Southeastern US, respectively. This reveals an ongoing and complex history for subsp. *fastidiosa* involving multiple introductions from diverse source populations.

On the other hand, the close relationship between Italian and Costa Rican subsp. *pauca* isolates indicates a likely introduction from the latter via infected plant material [79]. This hypothesis is based on the presence of the ST53 clade, which is so far absent from other populations on the American continent [18, 84], and is further validated here with whole genome sequence data. Subsp. *pauca* isolates from Brazil form a distinct monophyletic clade, with longer branch lengths showing that this is the most genetically diverse population for that subspecies. The current tree topology does not clearly establish the subsp. *pauca* population in Brazil as ancestral to the Costa Rican and Italian populations. Nonetheless, longer branch lengths suggest that population-specific events have led to increased diversification within Brazilian subsp. *pauca*. Previous studies suggested that the putative accumulation of beneficial and neutral mutations as well as the extended action of recombination between citrus-infecting and coffee-infecting lineages are drivers for the genetic diversity of Brazilian subsp. *pauca* [23].

While the present study attempted to represent *X. fastidiosa*’s diversity in Central and South America, our sampling of the region remains very limited. More thorough sampling has been done in only a few countries and it is largely aimed at plants of commercial or ornamental interest. Further sampling of native plants and in new locations would be necessary to fully establish the diversity of *X. fastidiosa*. Nonetheless, we expect that this study has contributed in highlighting the importance of the region in *X. fastidiosa* evolution and its global dispersal dynamics.

## Conclusions

The present study shows that the Central America *X. fastidiosa* populations have played an important role in the evolution of *X. fastidiosa* worldwide. Our results support the hypothesis that subsp. *fastidiosa* is native to Central America, from where it was introduced to North America, eventually these strains evolved and adapted to their new environment. Multiple gain/loss events occurring at an inter- and intra-subspecific level indicate that the genomes of *X. fastidiosa* subspecies found within Costa Rica are highly malleable. Furthermore, we found that compared to recent introduction events, native *X. fastidiosa* populations presented higher gene gain/loss, genome-wide recombination, and complex linkage disequilibrium patterns. This suggests that a combination of non-adaptive and adaptive forces (e.g. recombination followed by a selective sweep) might be key to *X. fastidiosa* host-switching and host adaptation. This is further supported by the lack of successful infection of *V. labrusca* by numerous and diverse Costa Rican strains. At the same time, the genetic diversity and ecological history of potential *X. fastidiosa* hosts has a crucial role in determining the genetic structure of the bacterial population, particularly in the case of monoculture crops. It has been recognized that the treatment, management, and control strategies for bacterial pathogens benefits from a better understanding of the ecological and evolutionary mechanisms leading to host adaptation [2]. Overall, the present study further shows the role that combined ecological and evolutionary events have in the development of plant disease and how their effects on a single population (i.e. Costa Rica) influences the evolutionary outcome of a world-wide pathogen such as *X. fastidiosa*.

## Supplementary information

**Additional file 1: Supplementary Table 1.** Metadata of *X. fastidiosa* isolates included on the present study.

**Additional file 2: Supplementary Table 2.** List of gene presence/absence of known virulence genes in *X. fastidiosa* subsp. *fastidiosa*, *X. fastidiosa* subsp. *pauca*, and subsp. *fastidiosa* and *pauca* isolates originating from Costa Rica.

**Additional file 3: Supplementary Table 3.** DAVID’s functional enrichment analysis for genes found within ancestral or recent recombinant regions.

**Additional file 4: Supplementary Table 4.** Summary of grapevine inoculation results from *X. fastidiosa* subsp. *fastidiosa* isolates in Costa Rica.

**Additional file 5: Supplementary Figure 1.** Heatmap showing gene presence/absence data on distinct functional gene categories. Roary’s binary gene presence/absence file was used to stablish gene presence (1) or absence (0). Phylogenetic trees show the relationship among subsp. *fastidiosa* and subsp. *pauca* isolates. The heatmap shows gene absence (red) and gene presence (blue) in relation to all evaluated isolates. Z-scores show the number of standard deviations below or above the population mean for each data point. Dark blue/red indicates gene presence/absence on few genomes. Lighter blue/red indicates gene presence/absence on several genomes. **a.** Genes belonging to the ‘Metabolism’ category; **b.** Genes belonging to the ‘Information storage and processing’ category; **c.** Genes belonging to the ‘Cellular processes and signaling’ category; **d.** Genes belonging to the ‘Uncharacterized’ category.

**Additional file 6: Supplementary Figure 2.** GLOOME’s tree branch length with gain/loss. Branch length are proportional to the total number of gain and loss events. Individual branch lengths are shown on the tree with line thickness being proportional to the number of gain/loss events on the corresponding branch. **a.** Tree with branch length determined by gain events (red branches). **b.** Tree with branch length determined by loss events (blue branches).

**Additional file 7: Supplementary Figure 3.** Ancestral recombination of two *X. fastidiosa* subspecies in Costa Rica. Colors indicate phylogenetically distinct *X. fastidiosa* lineages: *X. fastidiosa* subsp*. pauca* (red), *X. fastidiosa* subsp*. fastidiosa* (group 1, blue), and *X. fastidiosa* subsp*. fastidiosa* (group 2, green). **a.** Circular plot of strain-specific recombinant events. Each line represents a recombinant event, with the width and placement of the line indicating recombinant segment size and alignment position; **b.** FastGEAR recombination plot showing donor/recipient recombinant sequences and the position of the recombinant event in the alignment; and **c.** Heatmap showing the number of donor/recipient interactions among strains.

**Additional file 8: Supplementary Figure 4.** Placement of ‘unknown’ lineages identified in recent recombination events within Costa Rica. Each ML tree corresponds to an individual recombinant segment between an identified *X. fastidiosa* isolate and an ‘unknown’ lineage. Isolates recipient to the 'unknown' lineage are identified by colored bolded fonts. The ‘unknown’ sequences ancestral to other *X. fastidiosa* subsp. *fastidiosa* isolates are shown in red. The ‘unknown’ sequences from a recently divergent group within *X. fastidiosa* subsp. *fastidiosa* are shown in purple.

**Additional file 9: Supplementary Figure 5.** Location of 'unknown' lineages identified in recent recombination events within the complete dataset. Each ML tree corresponds to an individual recombinant segment detected between an identified *X. fastidiosa* isolate from Costa Rica and an ‘unknown’ lineage located within the complete dataset. Isolates recipient to the 'unknown' lineage are identified by colored bolded fonts. The ‘unknown’ sequences clustered within subsp. *fastidiosa* are shown in purple. The ‘unknown’ sequences clustered within subsp. *pauca* are shown in green. The ‘unknown’ that were ancestral to *X. fastidiosa* subsp. *fastidiosa* and/or subsp. *pauca* are shown in red.

**Additional file 10: Supplementary Figure 6.** Nucleotide diversity (Tajima’s D) and recombinant events across the length of the core genome alignment. Populations in line plot are identified with different colors: subsp. *fastidiosa* in California (blue), subsp. *fastidiosa* in Costa Rica (orange), subsp. *fastidiosa* in Southeastern US (purple), subsp. *pauca* in Brazil (green), subsp. *pauca* in Italy (dark yellow). **a.***X. fastidiosa* subsp. *fastidiosa*: Line plot showing Tajima’s D values across the length of the core genome alignment and fastGEAR output showing the location of recombination events among identified clusters. The identity of populations given in each cluster is given in the left size of the plot. **b.***X. fastidiosa* subsp. *pauca*: Line plot showing Tajima’s D values across the length of the core genome alignment and fastGEAR output showing the location of recombination events among identified clusters. The identity of populations given in each cluster is given in the left size of the plot.

**Additional file 11: Supplementary Figure 7.** Linkage Disequilibrium (Rozas’ ZZ) and recombinant events across the length of the core genome alignment. Populations in line plot are identified with different colors: subsp. *fastidiosa* in California (blue), subsp. *fastidiosa* in Costa Rica (orange), subsp. *fastidiosa* in Southeastern US (purple), subsp. *pauca* in Brazil (green), subsp. *pauca* in Italy (dark yellow). **a.***X. fastidiosa* subsp. *fastidiosa*: Line plot showing Rozas’ ZZ values across the length of the core genome alignment and fastGEAR output showing the location of recombination events among identified clusters. The identity of populations given in each cluster is given in the left size of the plot. **b.***X. fastidiosa* subsp. *pauca*: Line plot showing Rozas’ ZZ values across the length of the core genome alignment and fastGEAR output showing the location of recombination events among identified clusters. The identity of populations given in each cluster is given in the left size of the plot.

**Additional file 12: Supplementary Figure 8.** Variations on Rozas’s ZZ index across functional categories. Variations of Rozas’s ZZ index in the ‘Information storage and processing’, ‘Cellular processes and signaling’, ‘Metabolism’, ‘Uncharacterized’, and ‘Multiple’ categories for *X. fastidiosa* subsp*. fastidiosa* in Costa Rica (orange, above) and *X. fastidiosa subsp. pauca* in Brazil (green, below).

## Data Availability

All raw reads and information regarding each strain have been submitted to NCBI under the following bioproject numbers: PRJNA576471 (Costa Rican isolates) and PRJNA576479 (Brazilian isolates).

## Abbreviations

LD: Linkage Disequilibrium
subsp. *fastidiosa*: *Xylella fastidiosa* subsp. *fastidiosa*
subsp. *multiplex*: *Xylella fastidiosa* subsp. *multiplex*
subsp. *pauca*: *Xylella fastidiosa* subsp. *pauca*
