# Supplementary figures and images for: Impacts of local population history and ecology on the evolution of a globally dispersed pathogen
Source: BMC Genomics. 2020 May 20;21:369. doi: 10.1186/s12864-020-06778-6 (PMC7238557; doi:10.1186/s12864-020-06778-6)

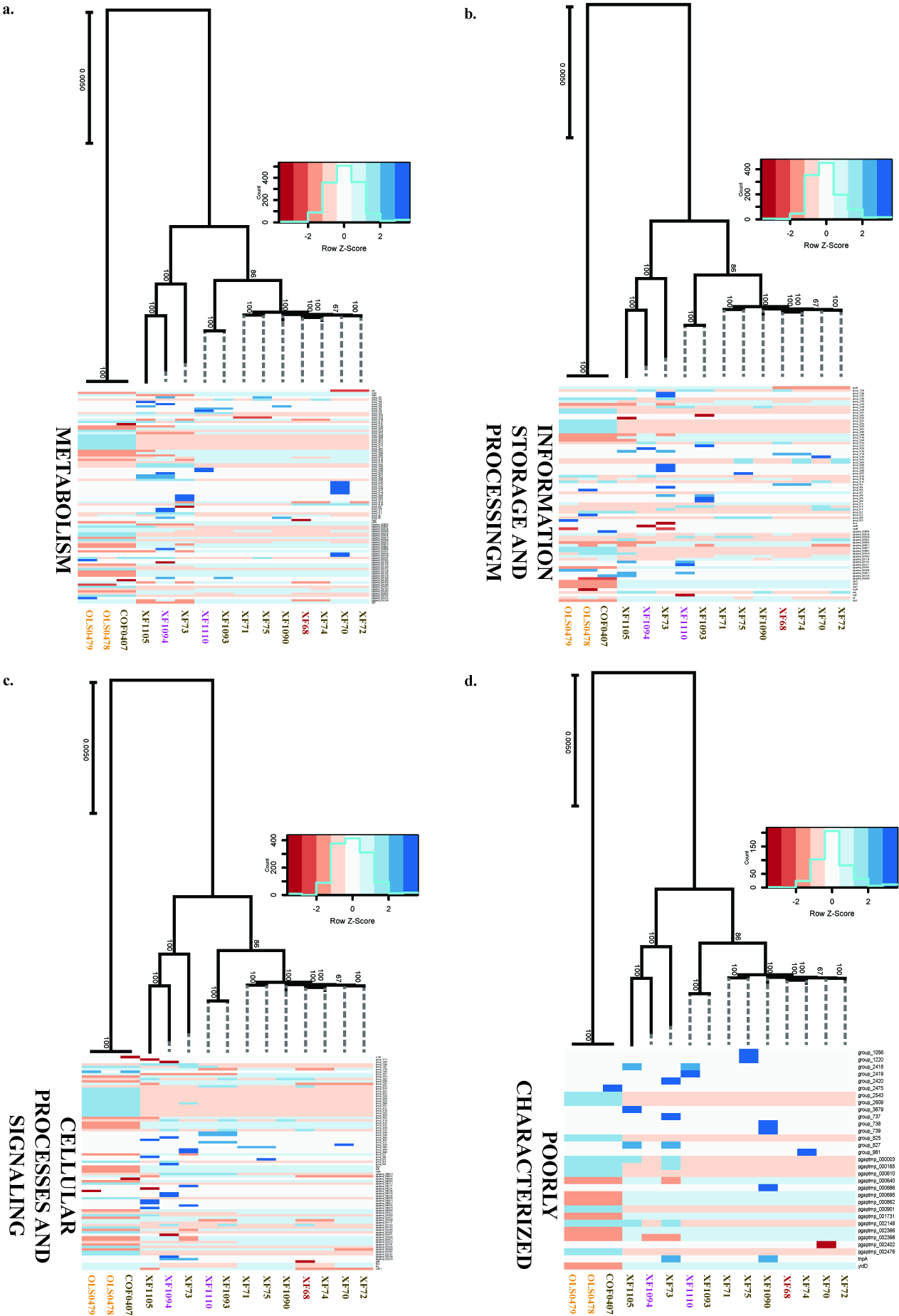

Supplement: Supplementary file 5 — Additional file 5: Supplementary Figure 1. Heatmap showing gene presence/absence data on distinct functional gene categories. Roary’s binary gene presence/absence file was used to stablish gene presence (1) or absence (0). Phylogenetic trees show the relationship among subsp. fastidiosa and subsp. pauca isolates. The heatmap shows gene absence (red) and gene presence (blue) in relation to all evaluated isolates. Z-scores show the number of standard deviations below or above the population mean for each data point. Dark blue/red indicates gene presence/absence on few genomes. Lighter blue/red indicates gene presence/absence on several genomes. a. Genes belonging to the ‘Metabolism’ category; b. Genes belonging to the ‘Information storage and processing’ category; c. Genes belonging to the ‘Cellular processes and signaling’ category; d. Genes belonging to the ‘Uncharacterized’ category. [file 12864_2020_6778_MOESM5_ESM.tif]

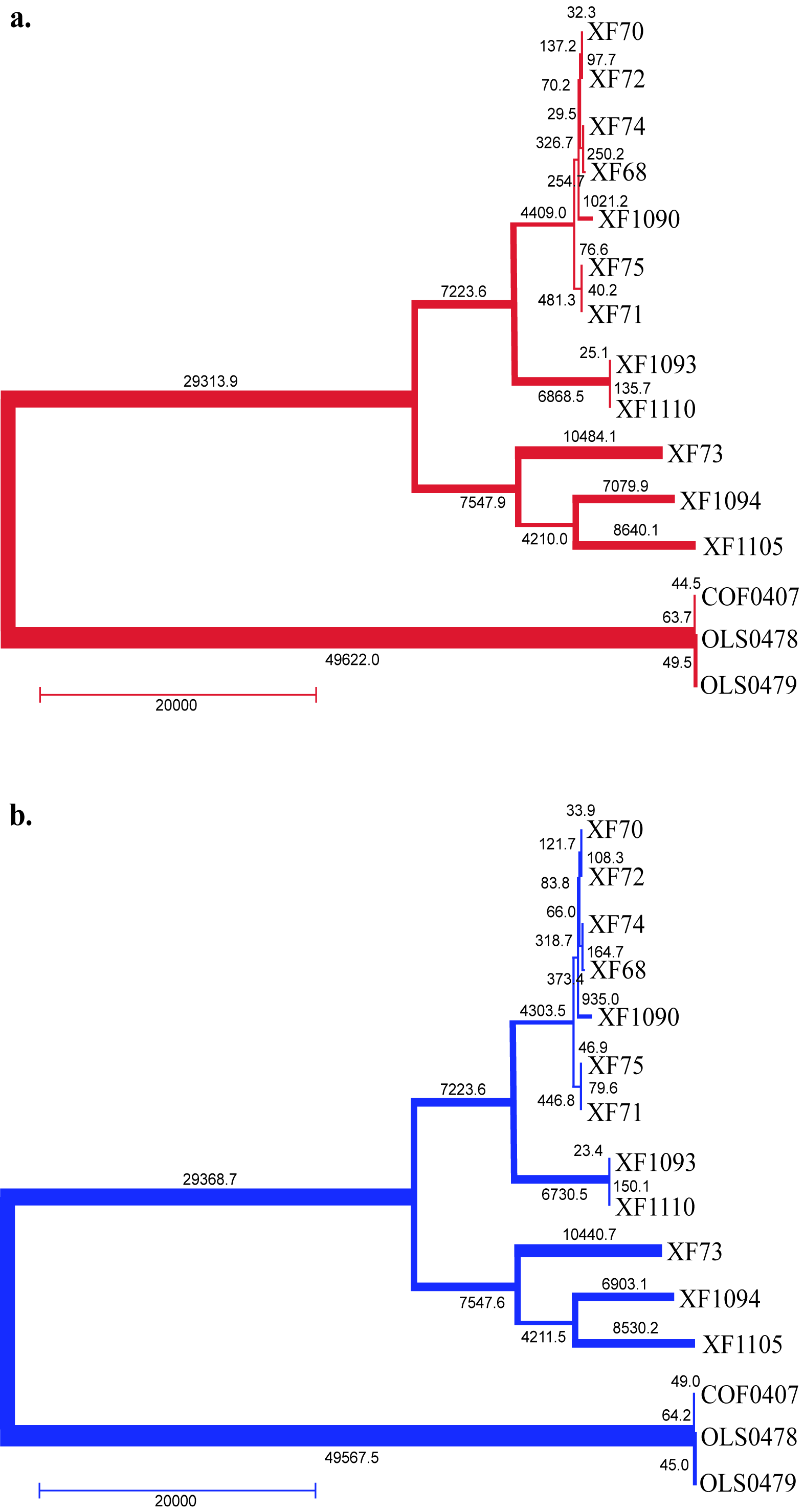

Supplement: Supplementary file 6 — Additional file 6: Supplementary Figure 2. GLOOME’s tree branch length with gain/loss. Branch length are proportional to the total number of gain and loss events. Individual branch lengths are shown on the tree with line thickness being proportional to the number of gain/loss events on the corresponding branch. a. Tree with branch length determined by gain events (red branches). b. Tree with branch length determined by loss events (blue branches). [file 12864_2020_6778_MOESM6_ESM.tif]

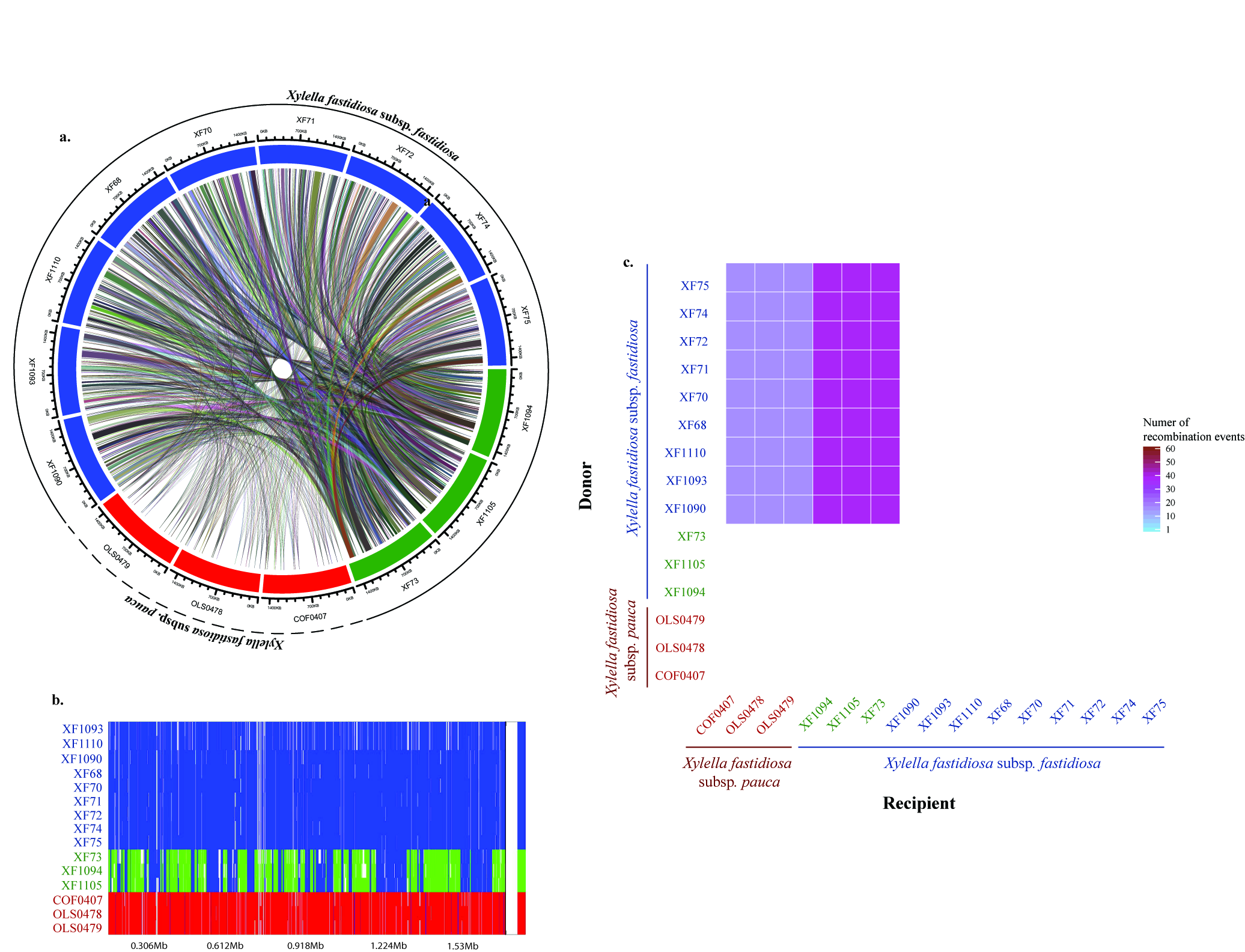

Supplement: Supplementary file 7 — Additional file 7: Supplementary Figure 3. Ancestral recombination of two X. fastidiosa subspecies in Costa Rica. Colors indicate phylogenetically distinct X. fastidiosa lineages: X. fastidiosa subsp. pauca (red), X. fastidiosa subsp. fastidiosa (group 1, blue), and X. fastidiosa subsp. fastidiosa (group 2, green). a. Circular plot of strain-specific recombinant events. Each line represents a recombinant event, with the width and placement of the line indicating recombinant segment size and alignment position; b. FastGEAR recombination plot showing donor/recipient recombinant sequences and the position of the recombinant event in the alignment; and c. Heatmap showing the number of donor/recipient interactions among strains. [file 12864_2020_6778_MOESM7_ESM.tif]

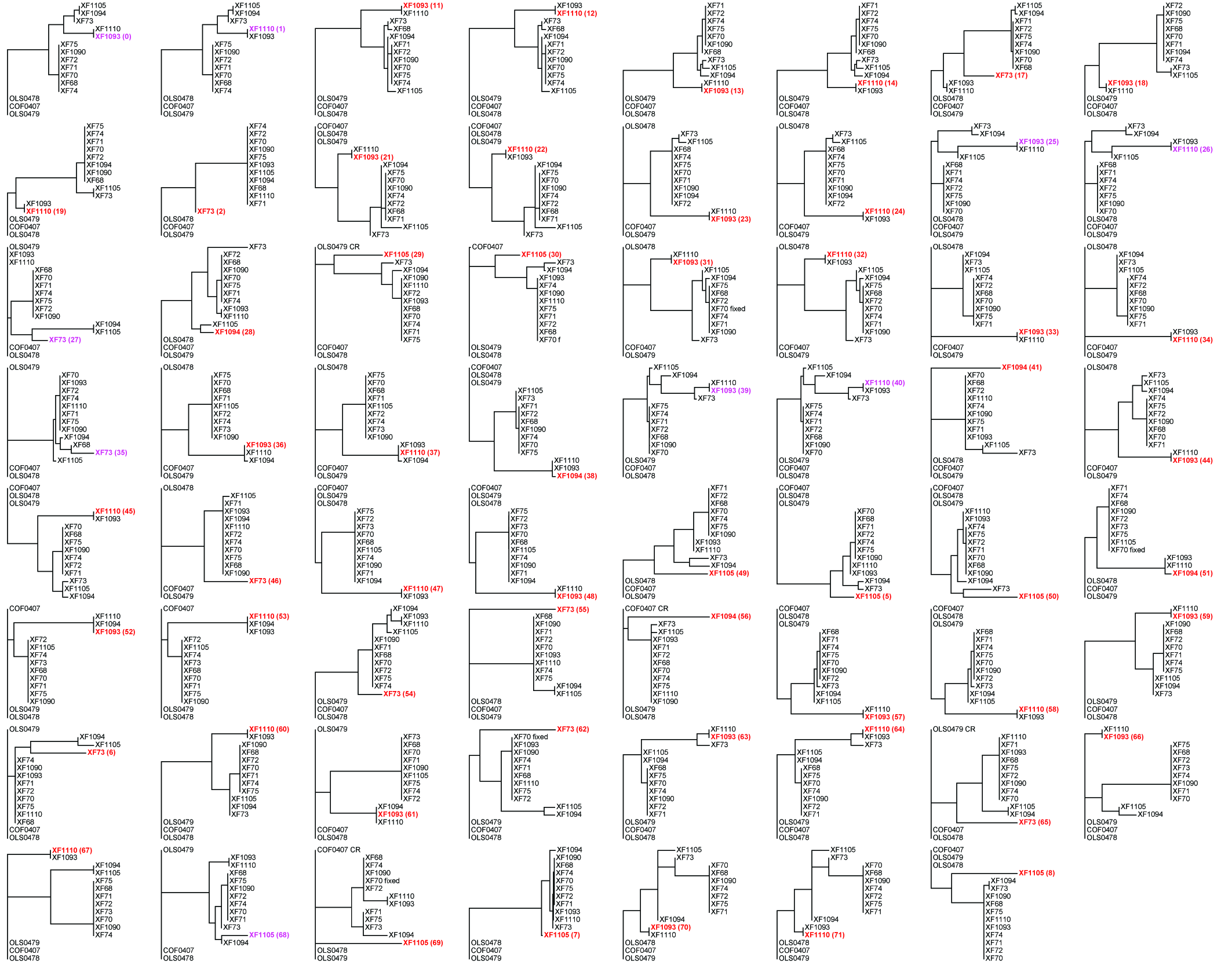

Supplement: Supplementary file 8 — Additional file 8: Supplementary Figure 4. Placement of ‘unknown’ lineages identified in recent recombination events within Costa Rica. Each ML tree corresponds to an individual recombinant segment between an identified X. fastidiosa isolate and an ‘unknown’ lineage. Isolates recipient to the 'unknown' lineage are identified by colored bolded fonts. The ‘unknown’ sequences ancestral to other X. fastidiosa subsp. fastidiosa isolates are shown in red. The ‘unknown’ sequences from a recently divergent group within X. fastidiosa subsp. fastidiosa are shown in purple. [file 12864_2020_6778_MOESM8_ESM.tif]

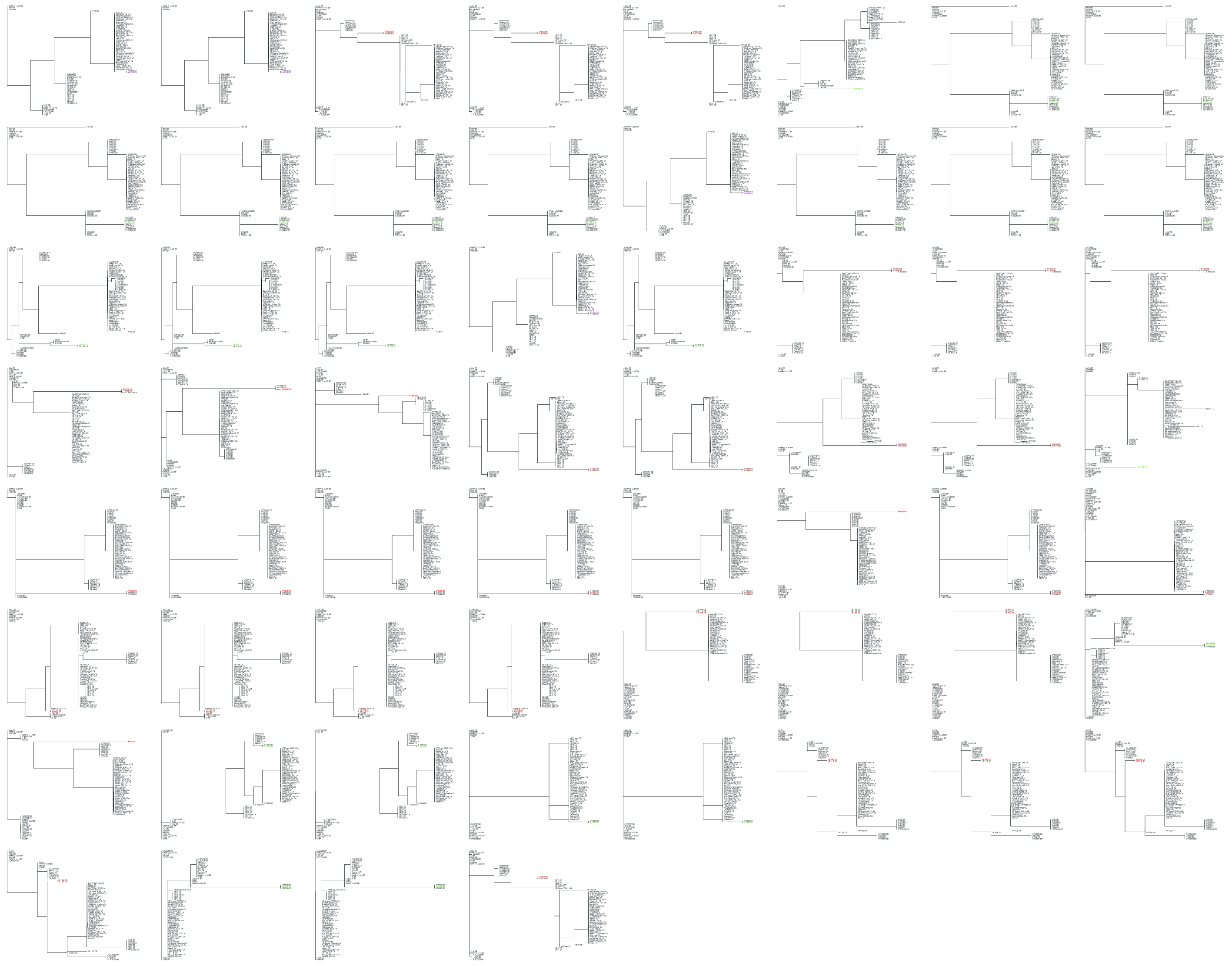

Supplement: Supplementary file 9 — Additional file 9: Supplementary Figure 5. Location of 'unknown' lineages identified in recent recombination events within the complete dataset. Each ML tree corresponds to an individual recombinant segment detected between an identified X. fastidiosa isolate from Costa Rica and an ‘unknown’ lineage located within the complete dataset. Isolates recipient to the 'unknown' lineage are identified by colored bolded fonts. The ‘unknown’ sequences clustered within subsp. fastidiosa are shown in purple. The ‘unknown’ sequences clustered within subsp. pauca are shown in green. The ‘unknown’ that were ancestral to X. fastidiosa subsp. fastidiosa and/or subsp. pauca are shown in red. [file 12864_2020_6778_MOESM9_ESM.tif]

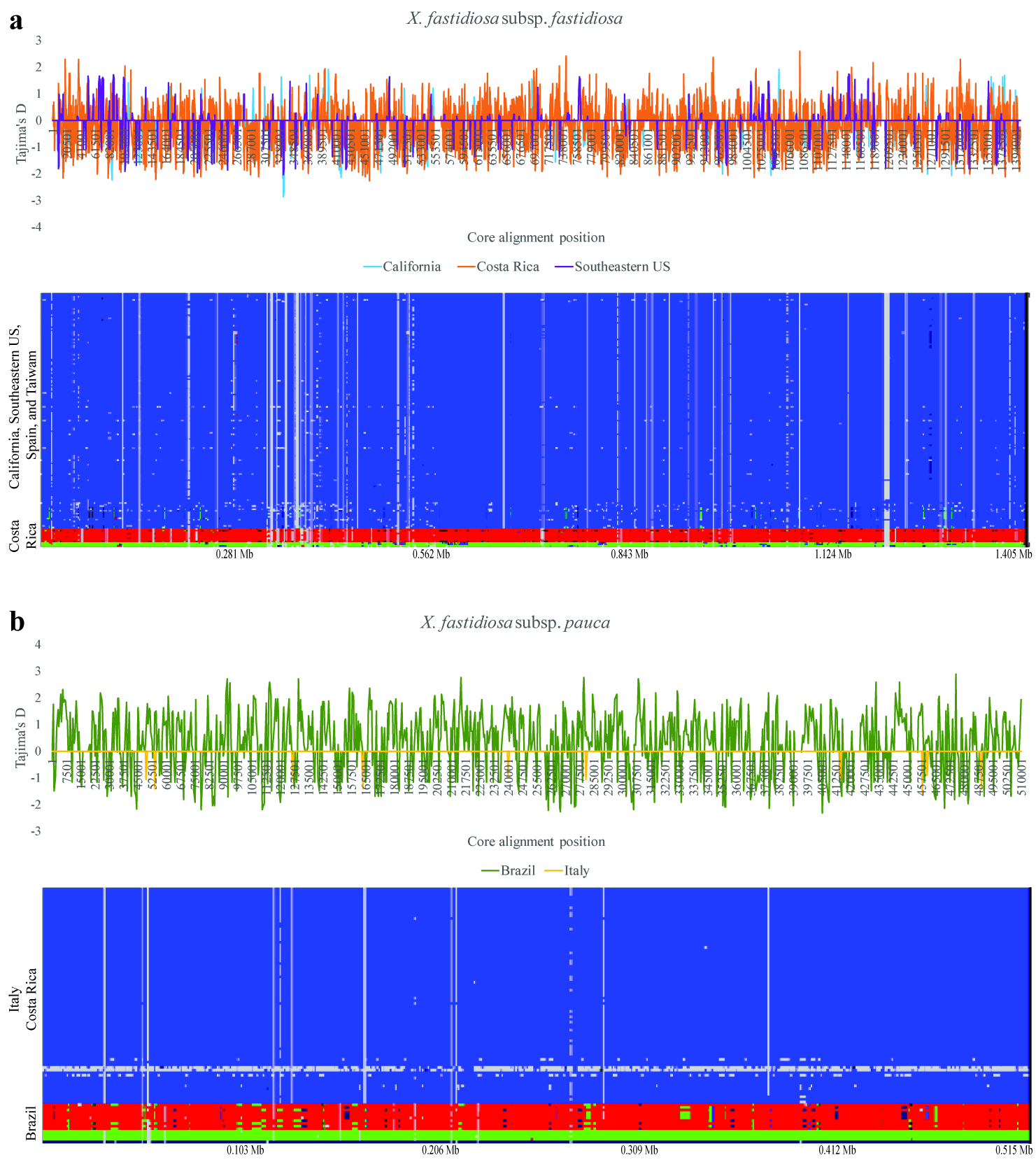

Supplement: Supplementary file 10 — Additional file 10: Supplementary Figure 6. Nucleotide diversity (Tajima’s D) and recombinant events across the length of the core genome alignment. Populations in line plot are identified with different colors: subsp. fastidiosa in California (blue), subsp. fastidiosa in Costa Rica (orange), subsp. fastidiosa in Southeastern US (purple), subsp. pauca in Brazil (green), subsp. pauca in Italy (dark yellow). a.X. fastidiosa subsp. fastidiosa: Line plot showing Tajima’s D values across the length of the core genome alignment and fastGEAR output showing the location of recombination events among identified clusters. The identity of populations given in each cluster is given in the left size of the plot. b.X. fastidiosa subsp. pauca: Line plot showing Tajima’s D values across the length of the core genome alignment and fastGEAR output showing the location of recombination events among identified clusters. The identity of populations given in each cluster is given in the left size of the plot. [file 12864_2020_6778_MOESM10_ESM.tif]

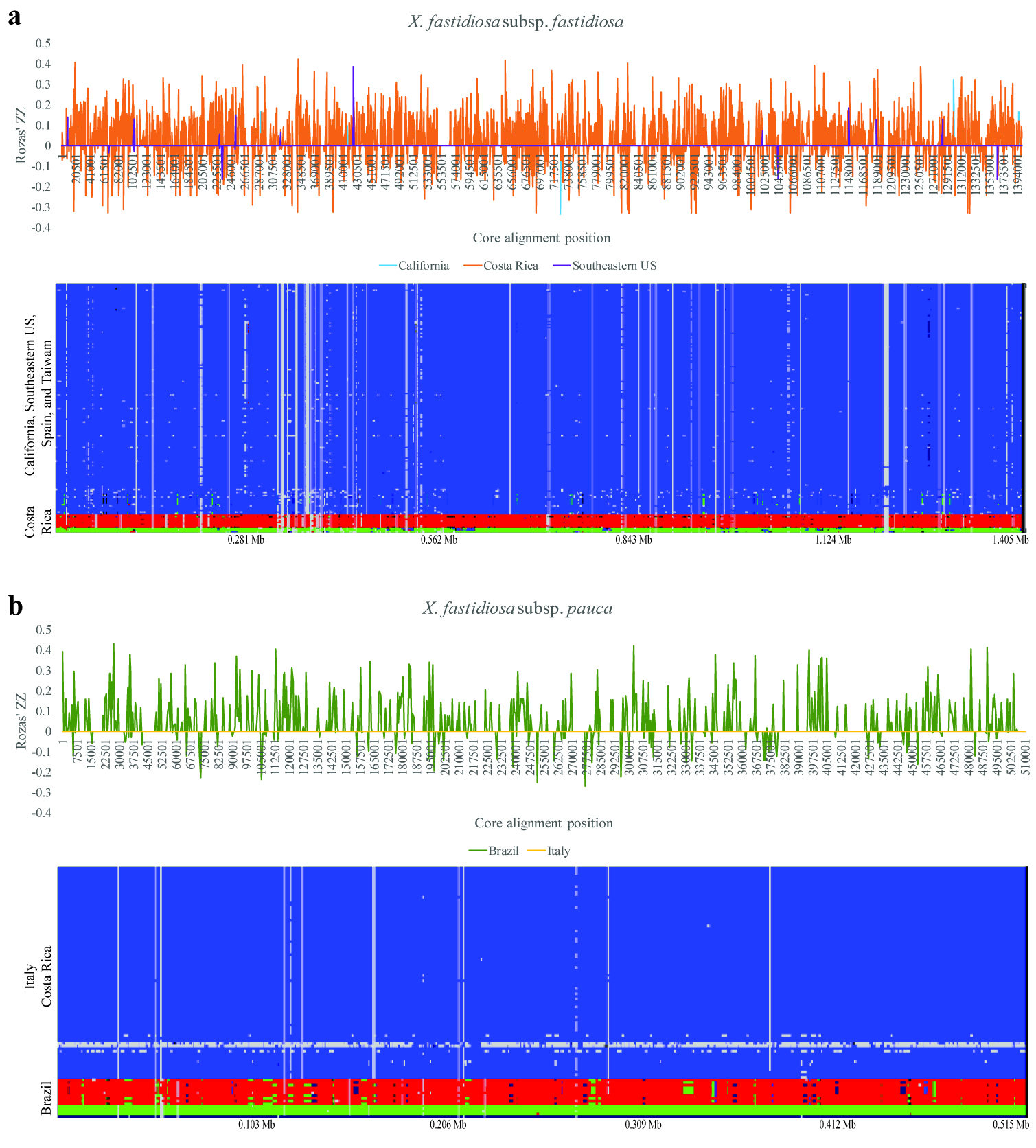

Supplement: Supplementary file 11 — Additional file 11: Supplementary Figure 7. Linkage Disequilibrium (Rozas’ ZZ) and recombinant events across the length of the core genome alignment. Populations in line plot are identified with different colors: subsp. fastidiosa in California (blue), subsp. fastidiosa in Costa Rica (orange), subsp. fastidiosa in Southeastern US (purple), subsp. pauca in Brazil (green), subsp. pauca in Italy (dark yellow). a.X. fastidiosa subsp. fastidiosa: Line plot showing Rozas’ ZZ values across the length of the core genome alignment and fastGEAR output showing the location of recombination events among identified clusters. The identity of populations given in each cluster is given in the left size of the plot. b.X. fastidiosa subsp. pauca: Line plot showing Rozas’ ZZ values across the length of the core genome alignment and fastGEAR output showing the location of recombination events among identified clusters. The identity of populations given in each cluster is given in the left size of the plot. [file 12864_2020_6778_MOESM11_ESM.tif]

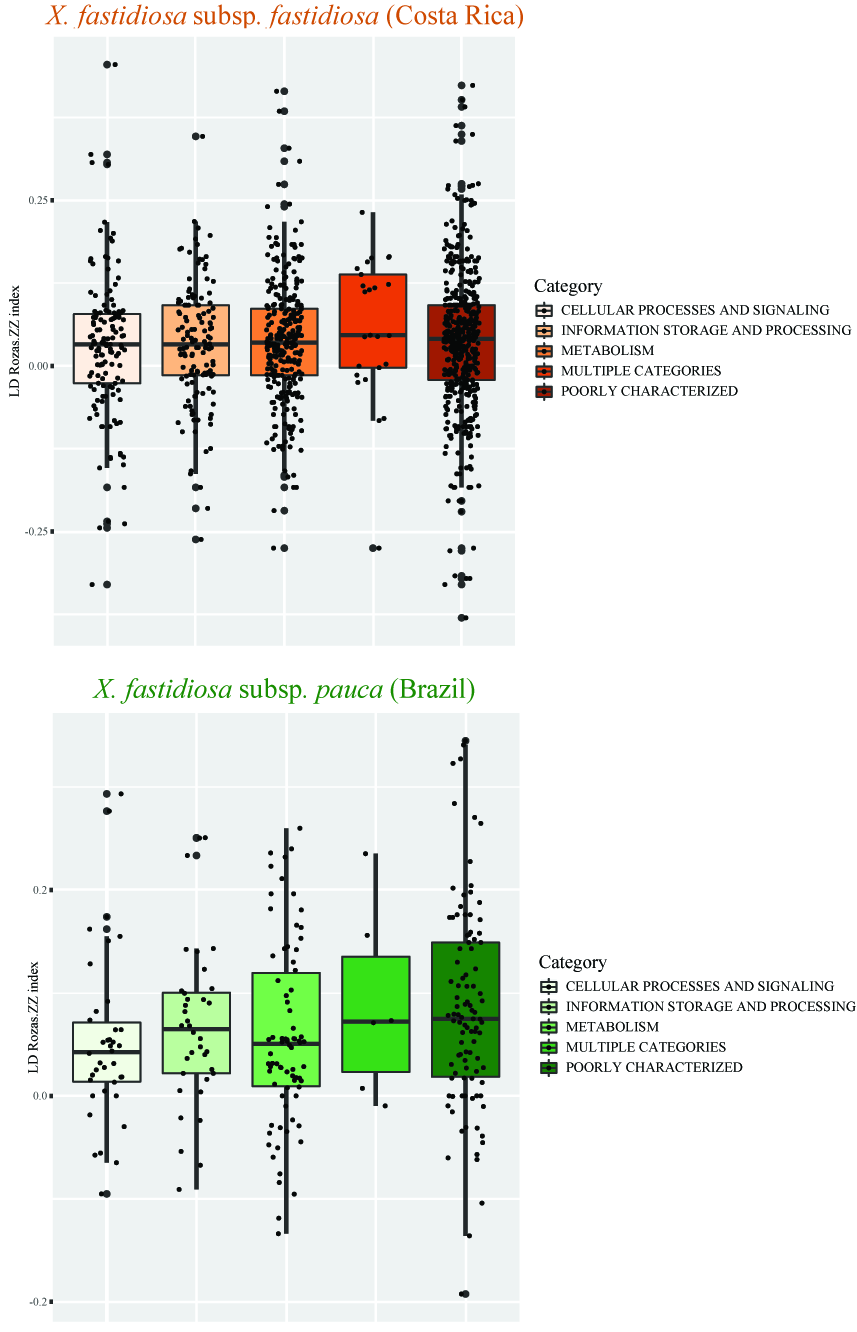

Supplement: Supplementary file 12 — Additional file 12: Supplementary Figure 8. Variations on Rozas’s ZZ index across functional categories. Variations of Rozas’s ZZ index in the ‘Information storage and processing’, ‘Cellular processes and signaling’, ‘Metabolism’, ‘Uncharacterized’, and ‘Multiple’ categories for X. fastidiosa subsp. fastidiosa in Costa Rica (orange, above) and X. fastidiosa subsp. pauca in Brazil (green, below). [file 12864_2020_6778_MOESM12_ESM.tif]
